# Magnetophoretic Micro‐Distributor for Controlled Clustering of Cells

**DOI:** 10.1002/advs.202103579

**Published:** 2021-12-15

**Authors:** Jonghwan Yoon, Yumin Kang, Hyeonseol Kim, Sri Ramulu Torati, Keonmok Kim, Byeonghwa Lim, CheolGi Kim

**Affiliations:** ^1^ Department of Emerging Materials Science DGIST Daegu 42988 Republic of Korea

**Keywords:** cell clustering, cell‐cell interaction, lab‐on‐a‐chip, magnetophoresis, single‐cell manipulation

## Abstract

Cell clustering techniques are important to produce artificial cell clusters for in vitro models of intercellular mechanisms at the single‐cell level. The analyses considering physical variables such as the shape and size of cells have been very limited. In addition, the precise manipulation of cells and control of the physical variables are still challenging. In this paper, a magnetophoretic device consisting of a trampoline micromagnet and active elements that enable the control of individual selective jumping motion and positioning of a micro‐object is proposed. Based on a numerical simulation under various conditions, automatic separation or selective clustering of micro‐objects according to their sizes is performed by parallel control and programmable manipulation. This method provides efficient control of the physical variables of cells and grouping of cells with the desired size and number, which can be a milestone for a better understanding of the intercellular dynamics between clustered cells at the single‐cell level for future cell‐on‐chip applications.

## Graphical abstract

The proposed method provides efficient control of the physical variables of cells and grouping of cells with the desired size and number, which can be a milestone for a better understanding of the intercellular dynamics between clustered cells at the single‐cell level for future cell‐on‐chip applications.

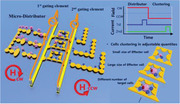

## 1 Introduction

The intercellular mechanisms or cell–cell interactions between cells, including junctions, phagocytosis, and pinocytosis, are crucial and fundamental functions for the analysis of cell differentiation, cancer metastasis, and immune mechanisms in various fields of biology and medicine.^[^ ^1^, ^2^ ^]^ For example, the understanding of the interactions between tumor cells and normal cells or reaction mechanism of tumor cells and immune cells at the individual cell level can enhance the realization of tumor development and metastasis, which can reveal more efficient prevention and safe treatment procedures.^[^ ^3^, ^4^, ^5^, ^6^, ^7^ ^]^ In general, immune cell responses are analyzed based on the averages of cell ensembles in various combinations of cell groups. However, variables such as heterogeneity between identical cells, different initiation times and contact periods, the number of interacting cells, and differences in antigen expression affect the immune response. It is challenging to determine the effects of these variables by measurements of ensemble groups.^[^ ^8^, ^9^, ^10^ ^]^

The physical properties of cells, such as size and shape, can result in biological heterogeneity that affects cell–cell interactions and rate and intensity of reactions in immune cells.^[^ ^11^, ^12^ ^]^ Therefore, control of physical variables of cells is required because of the distortion of individual characteristics at the single‐cell level and reduction in the confidence of the results obtained from the ensemble. Despite the need for variable control, fully satisfactory techniques have not been available to analyze the cell–cell interactions based on the physical properties of the cell, particularly microdevices that can cluster and monitor only the cells with the desired conditions. Thus, development of an efficient tool that can form a desired combination of cells by controlling the size and number of cells and monitoring the clustered cells is required.

Cell clustering methods have been developed to analyze and monitor interactions between cells. The cell‐clustering technology based on microfluidics has a high‐speed and high‐throughput function.^[^ ^13^, ^14^, ^15^, ^16^ ^]^ However, owing to the limited direction of motion caused by the unidirectional fluid flow, the flexible manipulation of individual cells is challenging, which hinders the selective control of the size and number of cells. Although the dielectrophoresis‐based cell‐clustering technology has the advantage of fluid‐free control of individual cells, it is challenging to control them by their physical parameters, such as the cell size and shape.^[^ ^17^, ^18^, ^19^ ^]^ However, the acoustic‐based cell‐clustering technology can control cells according to their physical factors, but it is challenging to control individual cells.^[^ ^20^, ^21^ ^]^ Hence, despite the advances in these technologies, the flexible manipulation of a single cell by physical parameters is challenging. Therefore, a simple technique that can manipulate and organize the desired cell combinations depending on the physical characteristics is required.

The magnetophoretic circuit can separate a large number of cells based on their size and shape, which can be manipulated individually by conjugation with various types of magnetic carrier bead in parallel.^[^ ^22^, ^23^, ^24^, ^25^, ^26^, ^27^, ^28^, ^29^, ^30^ ^]^ The movement of the magnetic carrier beads is determined by their physical properties, such as magnetism and size. This provides the most suitable technology for cell clustering. It can be freely accessible to a single cell‐level analysis device. Moreover, it can easily collaborate with other manipulated technology because it can be performed both in contradictory fluid environments such as completely closed or open‐channel structures. However, prior studies have only analyzed the dynamics of magnetic beads without considering the physical properties of micro‐objects or cells. Furthermore, the analysis of effect of micro‐objects on the dynamics of magnetic beads is challenging because dynamics was indirectly obtained through changes in local potential energy. These challenges of analysis can cause errors due to magnetic beads not being bound to cells in large amounts of automated cell analysis, and it can cause problems in experiments requiring sophisticated comparison groups. Consequently, those methods are not suitable for cell pairing applications, which require precise control of cell position according to the size and separation with high efficiency.

In this study, we develop an automated separation technique based on the size of micro‐objects and propose a highly integrated on‐chip management technology that enables selective clustering of cells based on their physical properties by using the parallel control and programmable manipulation of magnetophoretic circuits. The dynamic (jumping) motion of magnetic beads was analyzed through a numerical simulation of magnetic forces, which varied with the aspect ratio (AR) of the used ellipse‐shaped micromagnet and size of the micro‐object. At the optimized AR of the ellipse‐shaped micromagnet and frequency of the magnetic field for separation of micro‐objects with the desired size, the micro‐objects with a size difference of 1–2 µm can be separated by their size with high efficiency of 88.6%, including a fine separation of magnetically labeled THP‐1 cells according to their size differences. A micro‐distributor and clustering circuit are demonstrated using both target cell by number to isolate single effector cells and single effector cells by size through an active element.

## 2 Results and Discussion

### 2.1 Operation Principle of a Trampoline Micromagnet for Size‐Based Separation

In magnetophoretic circuits, magnetic carrier beads are trapped in a potential well created at the periphery of a micromagnet under a magnetic field and move according to the temporal changes with the rotating magnetic field. In particular, when the frequency of the external magnetic field increases and the movement of the potential well is accelerated, the viscosity resistance of the fluid pushes the beads in the opposite direction of the potential well, resulting in a position difference between the beads and potential well. This difference in position can be represented by the angular difference between the direction of the external magnetic field and the direction of the beads, referred to as phase lag (Figure S1a, Supporting Information). When the phase lag is above a certain critical value and the beads deviate from the potential well, the beads receive a repulsive force from the micromagnet.^[^ ^23^ ^]^ At this point, the beads exhibit a jumping motion away from the micromagnet. The magnetic force and viscous resistance influence the jumping motion. It is a random motion that can occur at any position on the disk‐shaped micromagnet owing to the same geometrical distribution of magnetic force in all magnetization directions.

In comparison, the ellipse‐shaped micromagnet has an AR (*b*/*a*) larger than 1, which increases the magnetic force in the *b*‐axis direction but decreases the force in the *a*‐axis direction. Therefore, the spatial magnetic force can be precisely controlled through the AR of the ellipse‐shaped micromagnet to control the position and height of the jump (Figure S1b, Supporting Information).

**Figure 1:**
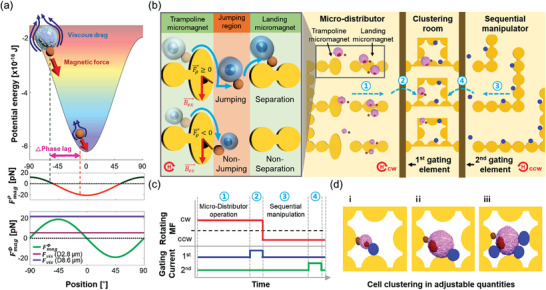
Working principle of micro‐distributor circuit contained trampoline micromagnet for cells clustering. a) Comparison of the magnetic force and viscous drag force applied to the micro‐objects. The micro‐object conjugated magnetic bead is located at a different position along with the potential energy well, and thus induces a bigger phase lag than the magnetic bead itself. Hence, the micro‐objects receive a bigger viscous drag force than magnetic force, and thus, they jump from the trampoline micromagnet due to *F* *_mag_* ^*ρ*^ . b) Schematic of the total micro‐distributor circuit for cell clustering which consists of a micro‐distributor, clustering room, sequential manipulator, and two gating elements. Again the micro‐distributor is composed of trampoline micromagnet and landing micromagnet. Micro‐objects receive positive*F* *_mag_* ^*ρ*^ from trampoline micromagnet for phase lag with size of the micro‐object and particular AR so that they can be separated to landing micromagnet through jumping and the micro‐distributor can be used as a size‐based separation magnetophoretic device (①), and a single micro‐object is isolated in the clustering room by 1st gating active element (②). After that, clustering group can be generated by transferring other micro‐objects from sequential manipulation segment using programable current on 2nd gating element (③‐④). c) Programmable manipulation process composed of rotating magnetic field and local current using gating element. As marked with numbers in a certain position in the image (b), each part of the proposed circuit is operated in order of under a clockwise and counter‐clockwise magnetic field. d) Isolation of adjustable numbers of micro‐objects (cells) with different clustering conditions. The clustering can be performed either by controlling the size of the micro‐objects (i and ii) or by controlling the number of micro‐objects (iii).

The immuno‐magnetically conjugated non‐magnetic micro‐objects, including cells, can be separated by viscous resistance depending on the size of the micro‐object, even if the same magnetic force is applied. Thus, as shown in **Figure** **1**a, the position of the beads depends on the size of the conjugated micro‐object as the potential well moves, which indicates that the phase lag is determined by the size of the micro‐object. Hence, micro‐object‐size‐controllable clustering can be performed in the micro‐distributor circuit, as shown in Figure 1b. The proposed circuit comprises a micro‐distributor for size‐based micro‐object separation, sequential manipulation segment, and gating elements, which control the number of micro‐objects and beads for clustering in the clustering room. The micro‐distributor comprises an ellipse‐shaped micromagnet, which resembles the jumping function of the mechanical trampoline. Thus, it is referred to as trampoline micromagnet and landing micromagnet for sorting of the micro‐objects. The phase lag and jumping distance of the micro‐objects were determined according to their size and AR of the trampoline micromagnet. The relatively large size of the micro‐object induces a larger phase lag than that for the small size of the micro‐object, with a jumping motion on the micromagnet with particular AR by repulsive force (*F* *_ρ_* ≥ 0).

Based on the analytical conditions, certain size of the micro‐object and AR of the micromagnet induce the jumping motion. Hence, the trampoline micromagnet can be used as a size‐based separation magnetophoretic device. Each segment of the device is operated by stages, marked with numbers in Figure 1b. Accordingly, micro‐objects are categorized by size on the landing micromagnet. A single micro‐object is isolated in the clustering room by the first gating active element under a clockwise rotating magnetic field. Clustering groups can be generated by transferring other micro‐objects from a sequential manipulation segment using the second gating element under a counter‐clockwise rotating magnetic field (Figure 1c). The size and number of micro‐objects can be precisely controlled by a programmable circuit. The desired variable clusters can be formed, as shown in Figure 1d.

### 2.2 Dynamic Manipulation of Magnetic Beads on the Trampoline Micromagnet

Depending on the rotation frequency of the external magnetic field, the motion of the magnetic bead is phase‐locked or phase‐slipping.^[^ ^22^ ^]^ The frequency at which the transition between these two modes occurs is referred to as critical frequency. When the micro‐object size and AR of the micromagnet increase, the critical frequency decreases as the phase lag increases at the same frequency. In addition, the change in the phase lag angle is caused not only by the frequency of the external magnetic field but also by the change in the micromagnet curvature (Figure S1b, Supporting Information). When the AR of the micromagnet increases, the curvature of the micromagnet changes along with the location of the micromagnet boundary. This change causes a noncontinuous alteration of the phase lag depending on the magnetic field direction with respect to the long‐axis direction of the elliptical micromagnet (Figure S1c, Supporting Information).

The boundary path of micromagnet can also lead to changes in the local speed of the magnetic bead. The path can be changed by AR or can be changed to a straight. When curvature is changed to straight, it is difficult to control the bead precisely due to the rapid change of movement of the bead, as shown in Figure S2 (Supporting Information). Therefore, the change according to AR has considered to analyze the local speed of magnetic beads.

**Figure 2:**
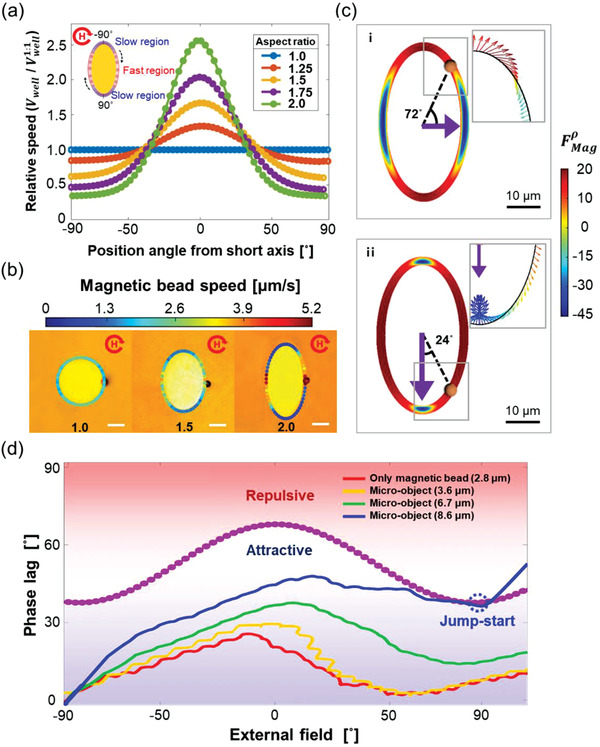
Dynamic manipulation of micro‐object on trampoline micromagnet. a) The difference in the speed of relative motion of the magnetic potential well depending on the AR of the trampoline micromagnet under the external rotating magnetic field. As the AR increases, the degree of relative variation of speed increases. (Insert shows the stroboscope image of the magnetic potential well along AR of 2 for the trampoline micromagnet under the rotation magnetic field). b) Instantaneous speed change of magnetic beads was measured according to the AR of trampoline micromagnet at low frequency (0.03 Hz, scale bar is 10 µm). Because of the change of potential well speed along the boundary of the micromagnet, the magnetic bead shows the different speeds at 0° direction and 90° direction at low and high curvature, respectively. c) Magnetic potential landscape according to the direction of the external magnetic field and insert shows the magnetic force distribution and intensity in the *ρ* direction (box area) and surroundings where the magnetic force strength is zero. The purple arrow is external magnetic field direction. When the phase lag exceeds the critical angle, the magnetic bead starts to receive repulsive force (outward direction arrow (red) indicates the repulsive direction and the strength is expressed with color). The certain angle between the dotted line and the purple arrow is a critical angle. When the field direction is 0°, the critical angle is 72° (i) which is larger than 24° (ii) due to the curvature difference. d) The critical angle where the attractive force (blue) and the repulsive force (red) intersect for a magnetic bead along the AR of 1.5 for trampoline micromagnet was plotted according to the external rotating magnetic field direction (Purple dotted line) and the phase lag changes were measured depending on micro‐object size (colored line) on the AR of 1.5 for trampoline micromagnet under 100 Oe rotating magnetic field. Phase lag increases as the micro‐object size increases and hence, 8.6 µm diameter of micro‐object can receive repulsive force and start to jump at the critical phase lag angle at 37°.

For example, the potential well movement on the trampoline micromagnet with an AR of 2 shows slow and fast regions in the large and small curvature areas, respectively, to a uniform external magnetic field speed (inset in **Figure** **2**a).

The change in the local speed of the relative potential well was calculated according to the AR based on the constant rotation speed of the applied magnetic field (Figure 2a). The relative speed at an AR of 2 were 0.35 and 2.5 times higher at 90° than at 0° from the short axis, respectively, with the largest curvature. The moving speed of a 2.8 µm magnetic bead on the trampoline micromagnet was measured at a low frequency (0.03 Hz) of the external rotating magnetic field, which was less influenced by the viscous drag force. The obtained speed trajectory of the magnetic beads was similar to that of the magnetic potential well (Figure 2b).

To understand the dynamics of the magnetic bead under various conditions, it is necessary to analyze the magnetic force exerted on the magnetic bead. The magnetic force can be expressed with three components in the tangential (*φ*) direction, radial (*ρ*) direction, toward the center of the micromagnet from the bead, and vertical (*z*) direction with respect to the micromagnet surface,^[^ ^30^, ^31^ ^]^

(1) $$\vec{F}=\frac{\chi_{v}V}{2\mu_{0}}\;\nabla \;\left(\vec{B}\cdot \vec{B}\right)=F_{\mathrm{mag}}^{\varphi }\;{\hat{e}}_{\varphi }+F_{\mathrm{mag}}^{\rho }{\hat{e}}_{\rho }+F_{\mathrm{mag}}^{z}{\hat{e}}_{z}$$

(2) $$\left\{\begin{array}{c} F_{\mathrm{mag}}^{\varphi }=\frac{\chi_{v}V}{\mu_{0}}\frac{1}{\rho }\left(B_{\rho }\frac{\partial B_{\rho }}{\partial \varphi }-B_{\varphi }\frac{\partial B_{\varphi }}{\partial \varphi }\right)\\ F_{\mathrm{mag}}^{\rho }=\frac{\chi_{v}V}{\mu_{0}}\left(B_{\rho }\frac{\partial B_{\rho }}{\partial \rho }+B_{\varphi }\frac{\partial B_{\varphi }}{\partial \rho }\right)\\ F_{\mathrm{mag}}^{z}=\frac{\chi_{v}V}{\mu_{0}}\left(B_{z}\frac{\partial B_{z}}{\partial z}\right) \end{array}\right.$$

where *χ_v_* is the magnetic susceptibility of the moving bead, *V* is the volume of the magnetic bead (*m* ^3^),  and *μ*_0_ is the permeability of vacuum (4*π*  ×  10^−7^N A^−2^).

When the magnetic bead moves along the micromagnet with a constant curvature (disk shape), it is under phase‐locked conditions with a constant balanced force without changing the magnetic forces. However, on a micromagnet with an uneven curvature, such as a trampoline micromagnet with an elliptical shape, the magnetic bead receives a different magnetic force that changes with the direction of the external rotating magnetic field. Therefore, the analysis of the variation in magnetic force strength in the curvature of the trampoline micromagnet according to the direction of the rotating magnetic field is very important for the optimization of size‐dependent sorting. However, if the magnetic field strength is small, the magnetic force can be distorted by multiple domains. To simplify the force due to the magnetic field orientation and curvature, we analyzed the rotating magnetic fields above 100 Oe, in which the multidomain effect becomes negligible.

In a trampoline micromagnet, the *φ*‐directional magnetic force controls the orbital motion of the micro‐object. Thus, if the frictional force between the substrate and micro‐object is larger than the *φ*‐direction magnetic force, the magnetic bead cannot rotate. Therefore, comparison between the *φ*‐directional magnetic force and frictional force is essential to understand the micro‐object dynamics. The *φ*‐directional magnetic force is minimal at 0° with the smallest curvature of the trampoline. Its strength decreases further as the AR increases. As shown in Figure S3 (Supporting Information) for the *φ*‐directional magnetic force and frictional force according to the AR, when the AR is higher than 3, the magnetic force is smaller than the friction force. Therefore, the magnetic bead cannot reach above 0° and continually stops at a fixed position regardless of the frequency of the rotating magnetic field. Therefore, the trampoline micromagnet can only act as a distributor device with an AR range of 1–3.

The *ρ*‐direction magnetic force dominates in the jumping motion of the micro‐objects. When the magnetic force in the *ρ*‐direction is directed toward the center of the trampoline micromagnet, the micro‐object receives an attractive force (*F*_*mag*_^*ρ*^ < 0) from the micromagnet, whereas, if the force direction is directed outward from the trampoline micromagnet, the magnetic force acts as a repulsive force ( *F* *_mag_* *^ρ^* ≥ 0), and thus the micro‐object jumps with the angle between the field and object position (Figure 2c). The *ρ*‐direction magnetic force changes the spatial distribution depending on the direction of the external magnetic field. Figure 2c shows the magnetic potential energy distribution, while the insets show the force distribution (box area with colored arrows) when the external magnetic field (purple arrow) is at 0° and 90°. The position where the magnetic force strength is zero is the intersection of the attractive and repulsive forces corresponding to the critical angle. The force depends on the curvature of the trampoline micromagnet. As the attractive force distribution region is narrowest at 90° with the largest curvature, a smaller critical angle is obtained for the applied field at 90° (Figure 2c i ) than at 0° (Figure 2c ii); the critical angle changes periodically.

To investigate the change in phase lag according to the size of the moving objects in the trampoline micromagnet, polystyrene beads with different diameters of 3.6, 6.7, and 8.6 µm were labeled with a magnetic bead. Under a rotating magnetic field with a frequency of 1.55 Hz, the phase lags at the three different sizes of the labeled micro‐objects were measured in a trampoline micromagnet with an AR of 1.5, as shown in Figure 2d (colored line). The purple symbol represents the critical angle. The upper region of the line corresponds to the repulsive force, while the lower region of the symbol line is the area dominated by the attractive force. The smallest angle is 37° when the external magnetic field is 90°. As expected, the increase in the viscous drag force, depending on the size of the micro‐objects, results in an increment in the phase lag. In particular, in the external field direction at 90°, the 8.6µm bead (blue line) shows the intersection of the phase lag angle and critical angle. As discussed earlier, the critical angle is smallest when the external magnetic field is at 90°. Based on this result, it is possible to predict the micro‐object jumping position and conditions on the trampoline micromagnet.

The dynamics of magnetic beads and magnetically labeled micro‐objects were experimentally analyzed on trampoline micromagnets according to the frequency of rotating magnetic fields with a strength of 100 Oe. Various trampoline micromagnets with ARs of 1.25 to 2.25 were used as micro‐distributors. The average velocity of the magnetic bead and micro‐object by their size was measured (Figure S4a, Supporting Information), as well as the critical frequency change and jumping distance according to the AR (Figure S4b, Supporting Information). In this dynamic analysis, the micro‐objects had different moving speeds and critical frequencies depending on their size. The critical frequency could also be adjusted by the AR of the trampoline micromagnet. This indicates that micro‐distributor devices can be used for various applications such as the selective separation of the desired size of micro‐objects or separation of micro‐objects with various sizes simultaneously by controlling the AR.

### 2.3 Optimization of the Micro‐Distributor for Size‐Based Separation

First of all, we optimized the size of the magnetic beads and micromagnet for 3–20 µm sized cell separation, generally is the single‐cell size range from a magnetic/physical perspective (Figure S5, Supporting Information). To perform autonomous separation of micro‐objects by size, the trajectory was simulated to predict the movement of the micro‐objects as a function of the fluid environment, rotating magnetic field speed, and micro‐object size based on the optimized micromagnet and bead.

The micro‐object movements in the fluid cause a viscous drag force. Hence, according to the Stokes’ law, the viscous drag force received by a micro‐object on a micromagnet surface be expressed as^[^ ^32^ ^]^

(3) $${\vec{F}}_{\mathrm{vis}}=\frac{6\pi \eta r\vec{v}}{\sigma \left(h\right)}\;\sigma \left(h\right)=1-\frac{9r}{16h}+\frac{r^{3}}{8h^{3}}-\frac{45r^{4}}{256h^{3}}-\frac{r^{5}}{16h^{3}}$$

where *η* (kg *m* ^−1^ *s* ^−1^) is the dynamic viscosity of the fluid,$\;\vec{v}$ is the instantaneous velocity of a micro‐object with a radius of *r*, and *h* is the distance of the micro‐object to the substrate.

(4) $$\vec{v}=\frac{F_{\mathrm{mag}}-F_{f}}{18\pi \eta r}$$

In this study, It is assumed that the micro‐object touches the substrate without lift forces effect, *h* is equal to *r* so that the *σ*(*h*)^−1^ is equivalent to 3. The micro‐object velocity in the steady‐state is obtained by balancing the magnetic force (*F_mag_*), viscous drag force (*F_vis_*), and friction force (*F_f_* = *F*_*Micro*−*object* *friction*_  + *F*_*bead* *friction*_) as shown in Equation (4), however, *F*_*Micro*−*object* *friction*_, gravitational, buoyancy, and lift force are negligible. (Figure S6 and Table S1, Supporting Information)

It is challenging to calculate the movement of the micro‐objects through analytical functions in a micro‐distributor because *F_mag_* by the magnetic bead received on the trampoline micromagnet continuously changes according to the position of the magnetic bead and phase lag with the direction of the external magnetic field. In the numerical simulation, the magnetic force can be decomposed into tangential (*F* *_mag_* ^*ϕ*^), radial (*F* *_mag_* ^*ρ*^),and vertical (*F* *_mag_* ^*z*^) (Equation 1). The micro‐object velocity (*v*(*ϕ*, *ρ*)) can also be decomposed into each direction. The motion of the micro‐object inside the magnetic field was then calculated. When (*t*  =  0) the tracking positions *ϕ*_*i*_,  *ρ*_*i*_ ( *i*  =  1, ⋅⋅⋅, *N*) are distributed uniformly around micromagnet, the tracking position *ϕ*_*i*_(*t*), *ρ*_*i*_(*t*) evolves according to

(5) $$\frac{d\vec{\varphi_{i}}}{\mathrm{dt}}\;=\frac{\;\vec{v}\left(\vec{\varphi_{i}}\right)}{\rho_{i}}=\frac{\;F_{\mathrm{mag}}^{\varphi }-\;\mu_{k}\;F_{\mathrm{mag}}^{z}}{18\pi \eta r\rho_{i}}$$

(6) $$\frac{d\vec{\rho_{i}}}{\mathrm{dt}}=\vec{v}\left(\vec{\rho_{i}}\right)\;=\;\frac{\;F_{\mathrm{mag}}^{\rho }-\;\mu_{k}F_{\mathrm{mag}}^{z}}{18\pi \eta r}$$

where *μ*_*k*_ is the friction coefficient between Streptavidin and Teflon‐coated substrate,^[^ ^33^ ^]^ where the micro‐object velocity (*v(ϕ* *_i_, ρ*_*i*_) is computed by Equations (5 and 6) by considering the local value of the magnetic force at the position *ϕ* *_i_, ρ*_*i*_ . The equation of motion of the tracking parts is integrated numerically in discrete time steps d*t* by employing a Verlet‐type integration algorithm^[^ ^34^ ^]^ using a software code.

**Figure 3:**
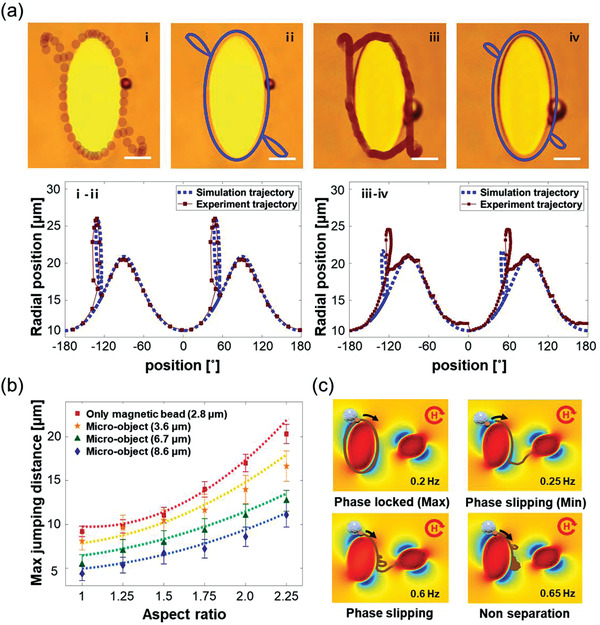
Comparison of the simulation and experimental trajectory and dynamics of the magnetic bead and micro‐object (8.6 µm) on the micro‐distributor. (a) Experimental (brown dotted line, i and ii) and numerically simulated (blue line, ii and iv) trajectories of magnetic beads and 8.6 µm diameter of micro‐object was implemented when they have two times jumping by entering the phase‐slipping regime on AR of 2 for of trampoline micromagnet (scale bar is 10 µm). The comparison of the experimental and simulated position of the magnetic beads (i‐ii) and micro‐object (8.6 µm diameter, iii‐iv), respectively, in radial coordinate with the center of the trampoline micromagnet set as the value of zero. b) Comparison of the simulation (dotted line) and experimental results of maximum jump distances according to micro‐objects size and AR of the trampoline micromagnet. (All experiments were averaged for 30 beads to obtain reliable statistics.) c) Trajectory simulation of 8.6 µm micro‐object on micro‐distributor composed of AR of 2 for trampoline micromagnet according to the frequency of rotating magnetic field. The micro‐object can be separated by jumping from trampoline micromagnet to landing micromagnet in the specific range of operating frequency, which is from 0.25 to 0.65 Hz. The gap between the landing magnet and trampoline micromagnet is 15 µm.

To validate the simulation, the experimental data were compared to the simulation results (**Figure** **3**a). A numerical simulation was carried out to calculate the frequency condition for an external rotating magnetic field with a strength of 100 Oe so that the bead jumps twice within one cycle. The experimental and simulated trajectories of the magnetic bead (**i, ii**) and micro‐object with a diameter of 8.6 µm (**iii, iv**) on the trampoline micromagnet with an AR of 2 were obtained using the calculated frequency parameter. The experimental trajectory under the same conditions is well‐matched with the simulated trajectory, as shown in **i‐iv**. Furthermore, the simulated maximum jumping distances at various sizes of micro‐objects according to the AR were compared to the experimental data. The jumping distance is the straight‐line distance of the micro‐object center from the boundary of the trampoline micromagnet. The maximum jumping distance increased with the decrease in the micro‐object size and increase in the AR, as shown in Figure 3b, which reveals a good agreement between them. Hence, the numerical simulation could provide design parameters for trampoline micromagnets that can automatically separate micro‐objects according to their sizes.

To separate the three types of micro‐objects, the micro‐distributor device should consist of three types of trampoline micromagnets and landing micromagnet to capture it after the jump. It is necessary to design the gap between the trampoline micromagnet and landing micromagnet by analyzing the influence of the magnetic force and potential energy distribution between the trampoline and landing micromagnet as well as the jumping distance of micro‐objects at a specific frequency. Therefore, we set the landing micromagnet with an AR of 1 and calculated the change in *F* *_mag_* ^*ρ*^ according to the gap between the trampoline micromagnet and landing micromagnet when the external magnetic field direction was at 0°. As shown in Figure S7a,b (Supporting Information), the *F* *_mag_* ^*ρ*^distribution around the trampoline micromagnet (AR = 2) and *F* *_mag_* ^*ρ*^ intensity according to the gap confirm that there is no significant mutual effect of the landing micromagnet when the gap is above 15 µm at a position of 7.5 µm away from the boundary of the trampoline micromagnet in the short‐axis direction. In this case, micro‐objects having jumping distances larger than 7.5 µm can only be separated into landing micromagnets. Based on the calculation, a separable gap was observed according to the AR and jumping distance. The largest micro‐objects (diameter of 8.6 µm) required the smallest gap size for separation. The maximum gap size for separation was calculated (Figure S7c, Supporting Information). Accordingly, a gap of 15 µm was evaluated to allow the separation function to be implemented regardless of AR. Numerical simulations on the separability conditions of three types of micro‐objects with diameters of 3.6, 6.7, and 8.6 µm were conducted with a gap of 15 µm between the trampoline micromagnets and landing micromagnets. The trajectory simulation results for a diameter of 6.7 µm on a trampoline micromagnet with an AR of 2 are shown in Figure 3c. According to the frequency, four types of dynamic trajectories are obtained. The micro‐object separation is possible from the critical frequency to below the nonseparation frequency.

**Figure 4:**
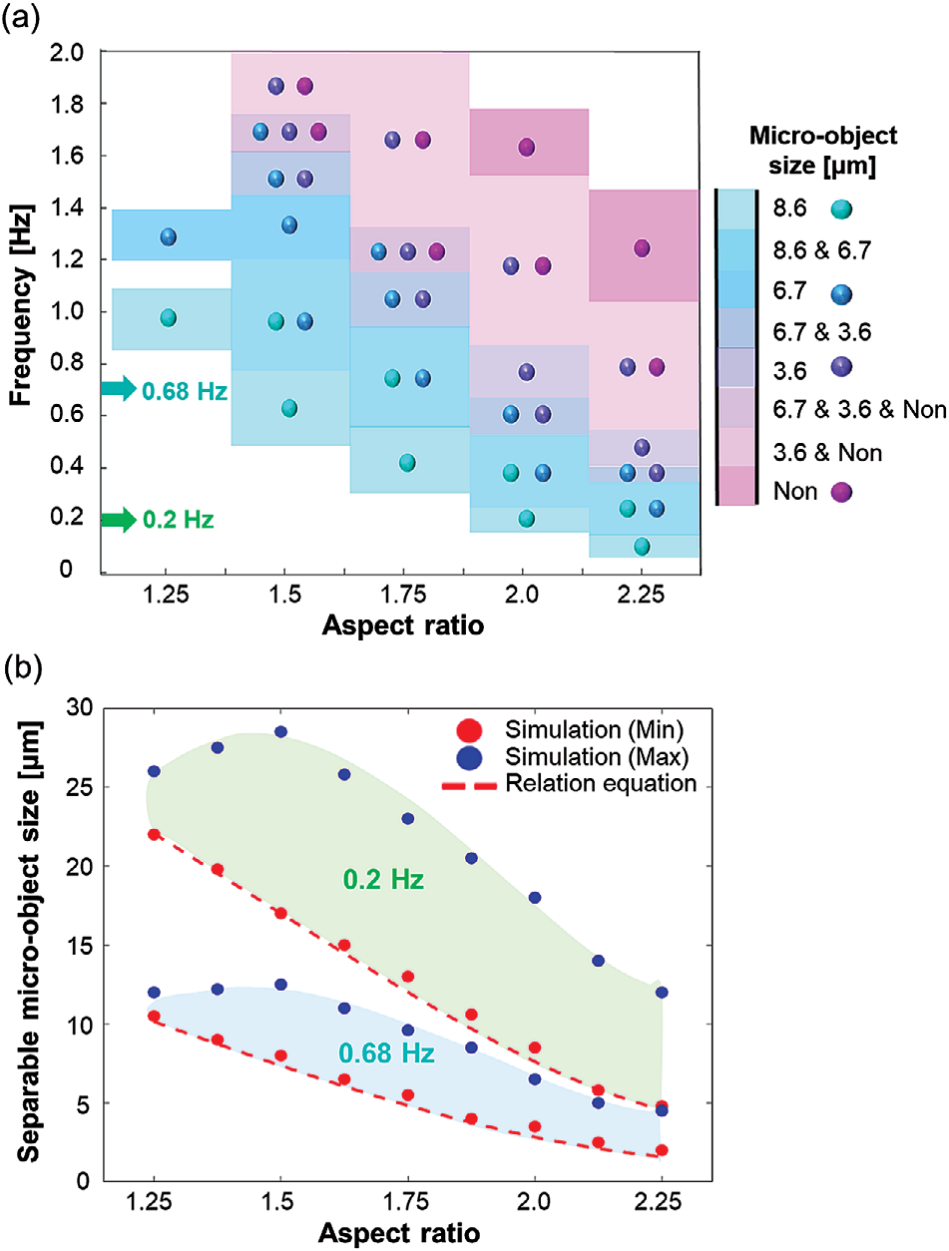
Phase diagram conditions for the size‐based separation of micro‐objects by micro‐distributor. a) The specific frequency region was calculated by numerical simulation according to the size of the micro‐object andAR by considering 15 µm of the gap between trampoline micromagnet and landing micromagnet. b) The region of separable micro‐object size according to AR of the trampoline micromagnet at 0.2 and 0.68 Hz frequencies. The red dots were simulation data of the minimum separable size of micro‐object and the blue dots were maximum separable size at a certain frequency. At 0.68 Hz, micro‐objects from 2 to 12 µm diameter range can be separated and from 5 to 25 µm diameter range of micro‐objects can be separated at the 0.2 Hz according to AR.

**Figure** **4**a shows the phase diagram, which indicates the frequency conditions of the external rotating magnetic field in which the three different sizes of micro‐objects (3.6, 6.7, and 8.6 µm) were separated according to the AR of the trampoline micromagnet using a trajectory simulation under various conditions.

When comparing the experimental results with the trajectory simulation included in the phase diagram, it can be seen that they are very consistent as shown in Figure S8 (Supporting Information).

According to the phase diagram, the micro‐distributor composed of three types of trampoline micromagnets with ARs of 1.5, 1.75, and 2 predicted that three types of micro‐objects with diameters smaller than 10 µm could be automatically separated by size within a rotating magnetic field at 100 Oe and 0.68 Hz. Hence, it can be confirmed that the operating conditions of the trampoline micromagnet were correlated with the AR, frequency, and size of the micro‐objects.

By formulating the correlation, critical frequency according to the size of micro‐objects and average viscous force were calculated based on Stoke's theory using the identified critical frequency and the size of micro‐objects. As shown in Figure S9 (Supporting Information) for micromagnet AR of 1, the separable viscous force has a constant value of 27.22pN (± 0.08). It is predicted that the separable viscous force varies according to AR.

As a result, the average viscous force on the micro‐object per one cycle of the external rotating field was obtained for different ARs (Figure S10a, Supporting Information). The relative formula can be expressed with minimum separable viscous force *F* *_vis_* ^*0*^ according to the AR as follows;

(7) $$F_{\mathrm{vis}-\mathrm{start}}\left(\mathrm{avg}\right)=\frac{F_{\mathrm{vis}}^{0}}{a\left(AR^{2}\right)+b\left(\mathrm{AR}\right)+c}\;,\;F_{\mathrm{vis}}^{0}=\;27.22\;\;\mathrm{pN}$$

The fitting constants were *a* = 4.53, *b* = −8.45, and *c* = 4.92.

Thus, the AR can be evaluated by the quadratic formula of Equation (8),

(8) $$AR_{\mathrm{mini}\mathrm{mum}}=\frac{-b+\sqrt{b^{2}-4a\left(c-\frac{F_{vis}^{0}}{36\pi^{2}\eta r\left(\rho_{\mathrm{pattern}}+r\right)f}\right)}}{2a}$$

*ρ*_pattern _is the radius of the trampoline micromagnet with AR of 1 and *f* is the frequency of the external field. Using this equation, the thresholds of separable ARs can be calculated according to the size of the micro‐object and frequency, as indicated by the dotted lines in Figure 4b. The blue and green regions indicate separable micro‐object sizes at 0.68 and 0.2 Hz. For example, to separate micro‐objects with dimensions of 12, 16, and 20 µm by size, trampoline micromagnets with ARs of 1.5, 1.75, and 2.125, respectively, can be automatically used at a frequency of 0.2 Hz. This allows to immediately design separable micro‐distributors according to the desired micro‐object size without simulating all conditions by finding a relational equation for the correlation between conditions.

**Figure 5:**
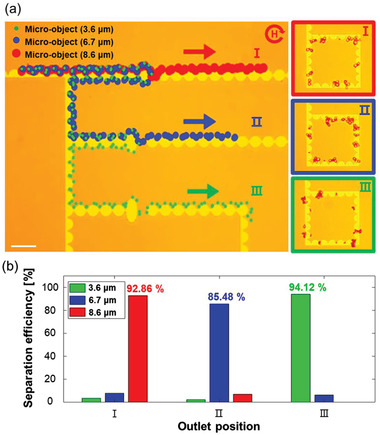
Automatic separation of micro‐objects. The separation of three different sizes of micro‐objects (3.6, 6.7, 8.6 µm) to the individual room was performed by the micro‐distributor with a gap of 15 µm. a) The trajectory of three sizes of micro‐objects on micro‐distributor and enlargement image of each room with resulting distribution of 175 complexes (*n*_3.6 µm_ = 85, *n*_6.7 µm_ = 62, *n*_8.6 µm_ = 28) of micro‐objects of three sizes. Separation experiments were implemented under 0.68 Hz and 100 Oe magnetic fields. According to the designed three types of trampoline micromagnet (AR: 1.5, 1.75, 2.0), micro‐object were separated sequentially from the largest micro‐object. b) The distributions ratios of thee sizes of micro‐objects were collected at each outlet position 1, 2, and 3 with an efficiency of 92.86%, 85.48%, and 94.12% (each separation efficiencies are calculated for n_I_ = 34, n_II_ = 57, n_III_ = 84 micro‐objects)

Hence, we devised a micro‐distributor device consisting of three types of trampoline micromagnets with ARs of 1.5, 1.75, and 2 and measured its efficiency by separating three types of micro‐objects with diameters of 3.6, 6.7, and 8.6 µm, respectively. Before the experiment, we constructed pre‐process circuit to select only one magnetic bead labeled micro‐object for demonstrating accurate size‐based separation (Figure S11, Supporting Information). The pre‐processed labeled micro‐objects mixture was loaded onto the collection part that is located at the front end of the micro‐distributor device. After loading, separation was performed for each micro‐object regardless of their initial position with a rotating magnetic field of 100 Oe at 0.68 Hz (Movie S1, Figure S12, Supporting Information). Three types of micro‐objects were sequentially separated from different trampoline micromagnets (**Figure** **5**a), composed of a pathway of loaded micro‐objects and isolated state in rooms 1, 2, 3 after the separation process. The micro‐objects with a diameter of 8.6 µm were separated into room 1 by jumping at an AR of the trampoline of 1.5, while smaller beads passed through it. Consecutively, the micro‐objects with diameters of 6.7 and 3.6 µm were separated into rooms 2 and 3, respectively. In the loaded mixture, the micro‐objects with diameters of 8.6, 6.7, and 3.6 µm accounted for 16%, 35%, and 49%, respectively. After separation, the three types of micro‐objects were isolated in rooms 1, 2, and 3, occupying 93%, 85%, and 94%, respectively. The micro‐distributor had an average separation efficiency of 88.86% (± 4.15) calculated from 9 times repeated experiment results (Table S2, Supporting Information), even for a small size difference of micro‐objects with 1–2 µm (Figure 5b).

### 2.4 Size‐Based Separation of THP‐1 Cells Using the Designed Micro‐Distributor

The ability to separate cells by size was demonstrated for live and dead THP‐1 cells from a cell solution. The reduction in the size of the cells killed by apoptosis compared to normal cells allows the distinction between dead and normal live cells.^[^ ^35^ ^]^ The size distributions of live and dead cells are presented by the red and blue lines in Figure S13 (Supporting Information), respectively. The live cells had a diameter of 15.1 (± 5.2) µm in 804 samples, while dead cells that died in reaction with Camptothecin,^[^ ^36^ ^]^ a drug that causes apoptosis volume decrease, had a diameter of 9.4 (± 6.5) µm in 374 samples. Before the experiment, a numerical simulation was carried out to predict separability conditions by the sizes of 8–20 µm (the THP‐1 size region) in the cell culture environment (10% FBS RPMI medium). Because the experimental environment was different from that of the previous micro‐object separation, the viscosity (*η*) was measured to be 0.0032 Pa s (Figure S14, Supporting Information) and the magnetic field strength was set to 130 Oe for a smooth motion with a high viscosity resistance.

**Figure 6:**
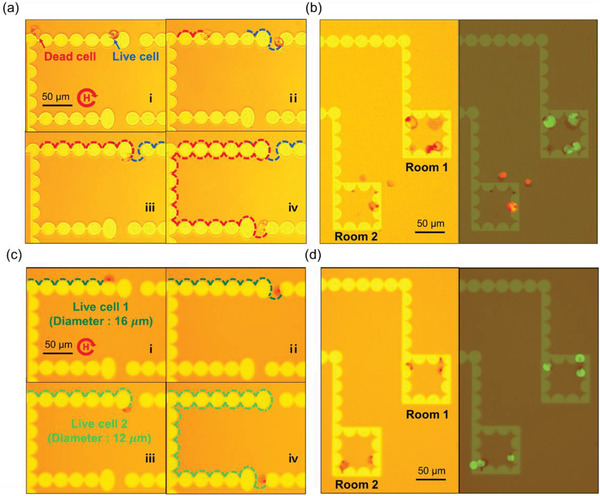
Simultaneous separation of live and dead cells. The separation of live and drug‐treated dead THP‐1 cells, and also the separation of different sizes of live THP‐1 cells was tested using a micro‐distributor in a cell culture environment (10 % FBS RPMI medium). a) The trajectory of live and drug‐treated dead THP‐1 cell on micro‐distributor (i‐iv). Based on the difference in sizes of live THP‐1 cells and drug‐treated dead THP‐1 cells, they are separated to each room under the rotating field of 130 Oe at 0.18 Hz frequency. b) Bright‐field and fluorescent images of the trapped live and dead single cells in the individual apartments (green: live, red: dead). c) Trajectory of different sizes of live THP‐1 cell on micro‐distributor (i‐iv). Based on the difference in live THP‐1 cells sizes caused by single‐cell heterogeneity, they are separated at each room under the rotating magnetic field of 130 Oe at 0.18 Hz frequency. d) Bright‐field and fluorescent images of the trapped live single cells in the individual apartments (green: live)

A phase diagram for the conditions of separation by the size of a single THP‐1 with a size range of 8–20 µm according to the AR is shown in Figure S15 (Supporting Information). The blue line indicates the region with a deviation of the size of live THP‐1 of 1*σ* (*σ* = 68.2%), while the green line indicates the region with a deviation of the size of dead THP‐1 cells of 1*σ*. The micro‐distributor was optimized for single‐cell size separation under cell culture environmental conditions based on the phase diagram. Live THP‐1 solutions and dead THP‐1 solutions were mixed to create cell solutions with various sizes and conjugated with magnetic beads with diameters of 2.8 µm. For the implementation of size‐based separation, the cell solution was loaded into the micro‐distributor that included two types of trampoline micromagnets with ARs of 1.25 and 1.5, designed based on the phase diagram. As shown in **Figure** **6**a,b, in the optimized micro‐distributor in an external rotating magnetic field of 130 Oe at 0.18 Hz, live single THP‐1 cells with diameters of 14–20 µm jumped at the first trampoline micromagnet with an AR of 1.25 and were isolated into room 1 (isolated cell size: 17.31 ± 1.76 µm). The dead single THP‐1 was separated into room 2 by jumping from the trampoline micromagnet with an AR of 1.5 (isolated cell size: 12.1 ± 1.23 µm) (Movie S2, Supporting Information). All cells were fluorescently dyed with calcein‐AM (green) and EthD‐1 (red) to confirm their survival (live: green, dead: red). In addition, the separation of live THP‐1 cells with different sizes is shown in Figure 6c,d. It was possible to detect differences in size due to cell heterogeneity and isolate the live THP‐1 solutions. Hence, the micro‐object separation and cell separation demonstrated that the micro‐distributors enable separation in a wide size range of 3 to 20 µm and that they can be freely designed and programmed for desired size ranges for separation purposes.

### 2.5 Demonstration of a Clustering Circuit

A controllable micro‐distributor circuit was devised by combining active elements and trampoline micromagnet, which can be used as a device for a cell–cell interaction analysis system that can trap single effector cells by size and clustering with target cells in desired numbers. Active elements allow the magnetic bead to be positioned between different micromagnets by applying a current.^[^ ^37^ ^]^ Therefore, the spatial separation and number of separated micro‐objects can be controlled after size separation using a trampoline micromagnet. Using a micro‐clustering circuit, the size and number of micro‐objects can be controlled and various combinations of the two variables can be effectively generated (Figure S16, Movie S3, Movie S4, Supporting Information).

**Figure 7:**
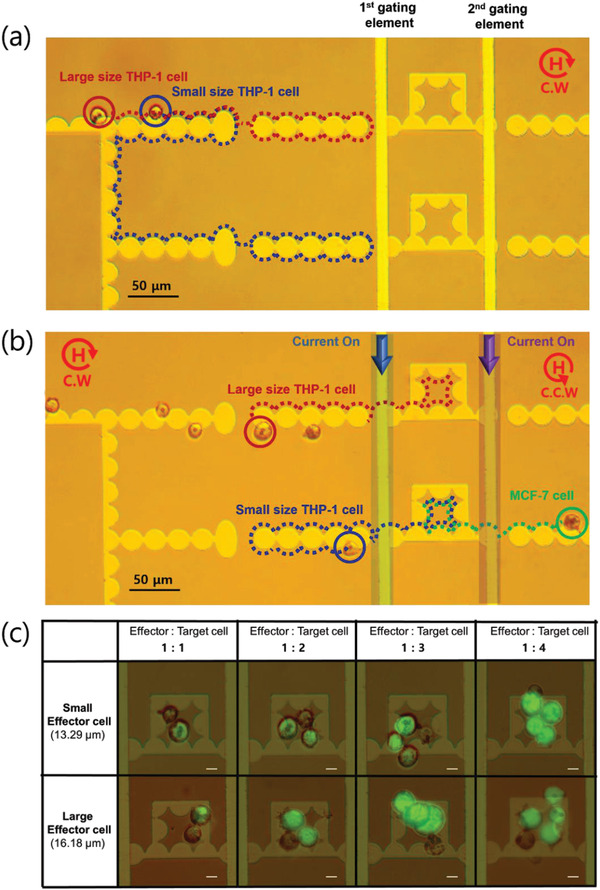
Cell clustering micro‐distributor performance. Different transition trajectories pathways were obtained for THP‐1 of different sizes considered as effector cells MCF‐7 considered as target cells using a micro‐distributor circuit for cells clustering (Movie S5). a) Size‐based distribution of micro‐objects was obtained at *f* = 0.18 Hz in a clockwise direction at 130 Oe magnetic field. On the micro‐distributor including AR of 1.5, 1. 75 of two types trampoline micromagnet, THP‐1s were separated to landing micromagnet for staying before applying local current. b) Selectively move only one THP‐1 which is distributed by size to each room by applying 75 mA of electric current (blue region) on the 1st gating element. After transferring one THP‐1 using current, when the magnetic field rotates counterclockwise, MCF‐7s was moved toward the current line. Then, MCF‐7 was programmable moved to each room by applying the time pulse current of 75 mA (purple region) to the 2nd gating element. c) A table of the various combination according to the size of THP‐1 and number of MCF‐7. The THP‐1 and MCF‐7 were considered as effector cells and target cells to demonstrate the performance of the cells clustering.

To demonstrate proof‐of‐principle of the cell clustering, magnetic bead labeled THP‐1 cells and MCF‐7 cells were used. The operation of the combinatorial function is illustrated in **Figure** **7** and Movie S5 (Supporting Information). In a clockwise‐rotation magnetic field, labeled THP‐1 cells (considered as effector cells) are separated by size through a micro‐distributor (Figure 7a). By applying an electric current through an active element, only a single micro‐object is transferred and trapped in an apartment (Figure 7b). Afterward, a counter‐clockwise‐rotation magnetic field is applied to turn off the separation in the micro‐distributor and move the labeled MCF‐7 cells (considered as a target cell), as shown in Figure 7b. When the current impulses, the target cells are quantitatively paired into each room, where a labeled single THP‐1 (effector cell) is trapped by size. As a proof‐of‐concept, the labeled MCF‐7 cells were then quantitatively separated into each apartment via a current impulse. The result is shown in the image table in Figure 7c for various combinations of the size‐based separation of labeled THP‐1 cell and quantitative separation of labeled MCF‐7 cells, which stained with green fluorescence. The apartment was designed by considering the actual size of the cells. Therefore, two or more cells can be sorted in the apartment and intercellular interactions can be observed because of the physical contact between cells.

## 3 Conclusions

A micro‐distributor circuit suitable for a cell interaction analysis system was developed, which consisted of a micro‐distributor based on a trampoline micromagnet and micro‐clustering device capable of quantitative distribution by using an active element at a specific location. The jumping motion of micro‐objects was analyzed by experiments and numerical simulations according to the size of the micro‐object and AR of the trampoline micromagnet. Through the correlation equation of the size of the micro‐object and AR, the circuit design was optimized and operating conditions that allow automatic separation of micro‐objects by size were obtained, where micro‐objects with sizes of 3 to 10 µm could be separated with a difference in size of ± 1 µm. The micro‐objects with three sizes (3.6, 6.7, 8.6 µm) coupled to magnetic beads were automatically separated with a high efficiency of 88.68%. In addition, live and dead cells were separated. The heterogeneity of cells by size was demonstrated for live THP‐1 cells in the range of 8–20 µm.

Micro‐objects separated by size were individually trapped in a room through the active element and clustered in various combinations by the micro‐distributor circuit. As a proof‐of–concept, cell clustering experiment was performed by using two types of cell to control the size and number of cells. By controlling the size of THP‐1 cells and number of MCF‐7 cells being clustered, various quantitative distributed combination groups could be created. Though, the proposed device was optimized to operate 3–20 µm size of micro‐objects, it can be designed to offer selective size‐based separation and clustering of various cell types which has larger (>20 µm) size regardless to cell weight or surface charge. In the case of transportable biomaterials of 1 µm or less, it is expected that control will not be possible because an appropriate level of magnetic force cannot be regulated by comparing the influence of Brownian motion.

In addition, if the bonding structure or the linker between a single cell and a bead is replaced with a cleavable structure, it is possible to remove the magnetic bead on clustered cells to eliminate its influence on cell itself.^[^ ^38^, ^39^, ^40^ ^]^ Therefore, the proposed device enables selective separation and quantitative clustering using programmable designs, which provide an efficient lab‐on‐a‐chip platform for intercellular interaction analysis, and it will be possible to use in various biomedical applications such as the identification of interactions between cells, drug reactivity, and cell engineering using a combination of cells implemented in the optimal environment.

## 4 Experimental Section

### Fabrication of Micromagnets and External Magnetic Field

The fabrication of micromagnets has been reported.^[^ ^24^ ^]^ Briefly, by photolithography and direct‐current magnetron sputtering, ellipse‐shaped micromagnets of a Ni_80_Fe_20_ thin film (thickness: 100 nm) were produced on a Si substrate. A Teflon film (thickness: 500 nm) was then coated to reduce the surface adhesion between beads and cells. Four ferrite core solenoid coils produced a rotating in‐plane magnetic field within the *x–y* plane. The rotating magnetic field strength and frequency were controlled using LabVIEW.

### Magnetic Simulation

The micromagnetic domain was simulated using the MuMax3 software. An exchange stiffness of *A*_ex_ = 1.0 × 10^−11^ J m^−1^, saturation magnetization of *M_s_* = 800 kA m^−1^, damping constant of *α* = 0.1, zero magnetocrystalline anisotropy, and cell dimensions of 100 × 100 × 100 nm^3^ were used for the simulation as standard material parameters. The distribution of the magnetic potential energy and magnetic force exerted on the bead were calculated based on the stray field obtained by the simulated magnetic domain using the Matlab code.

### Conjugation of Polystyrene Beads to Magnetic Microbeads

Superparamagnetic beads with diameters of 2.8 µm (M‐270 carboxylic acid, Invitrogen, Grand Island, NY, USA) were bound with nonmagnetic polystyrene beads with sizes of 3.6, 6.7, and 8.6 µm (aminopolystyrene beads, AP‐35‐10, AP‐60‐10, AP‐100‐10, SPHERO). The magnetic beads (5 µL) were washed with a 0.05 m MES (2‐(N‐morpholino) ethanesulfonic acid) buffer solution (pH 5) three times and added in 0.5 mL of a 0.05 m MES buffer solution. The magnetic beads, polystyrene beads (0.2% w/v), and EDC (*N*‐(3‐dimethylaminopropyl)‐*N*′‐ethylcarbodiimide hydrochloride, 50 × 10^−3^ m for polystyrene beads with diameters of 8.6 µm and 5 × 10^−3^ m for the other sizes of polystyrene beads) were added to a 0.05 m MES buffer solution with a total volume of 0.5 mL. The solution was incubated for 2–3 h with a slow tilt rotation. Subsequently, the resulting magnetic‐bead‐labeled polystyrene beads were separated using a magnet and washed with phosphate buffered saline (PBS) ( 0.04% Tween‐20). The conjugated beads were resuspended in deionized water for the experiment and micro‐objects with only one magnetic bead attached to the polystyrene bead was selectively used using a filtering micromagnet device (Figure S11, Supporting Information)

### Cell Culture and Induction of Apoptosis

THP‐1 (ATCC TIB‐202) cells MCF‐7 (ATCC HTB‐22) cells were cultured in a RPMI 1640 medium (Gibco 11875‐085) and DMEM (Gibco 12430‐054) supplemented with 10% FBS and 1% penicillin/streptomycin. Cell cultures were established using 2–4 × 10^5^ viable cells mL^−1^. To prepare THP‐1 dead cells, Camptothecin (Sigma Aldrich, C9911) was added to the cell solution (1 × 10^6^ cells mL^−1^) at a concentration of 50 × 10^−6^ M and incubated for 48 h.

### Conjugation of Antibodies to Magnetic Microbeads

Two types of antibody, HLA‐A2 and EpCAM, were used for labeling magnetic bead to THP‐1 and MCF‐7, respectively. Each antibody solution (0.5 mg mL^−1^) and Biotin‐PEG‐NHS linker solution (1 mg mL^−1^) were mixed using a vortex rotator for 1 h to obtain biotinylated antibodies. The solution was filtered using spin desalting columns to remove unbound linkers. Streptavidin coated magnetic beads (diameter: 2.8 µm) (M‐280; Invitrogen, Grand Island, NY, USA) were bound with biotinylated antibodies. The beads were washed with PBS (pH 7.4). Each biotinylated antibodies were added to 5 µL of the bead solution at a final antibody concentration of 3 × 10^−6^ g mL^−1^. The solution was well mixed using a vortex rotator for 2–3 h at room temperature. To remove any unbound antibodies, the beads were washed several times with PBS (0.02% Tween‐20) using a magnet. Finally, the antibody‐bound particles were resuspended in PBS (0.5 mL) and stored at 4 °C for further use.

### Binding of Cells to Antibody‐Coated Magnetic Microbeads

The cultured THP‐1 and MCF‐7 (1 × 10^6^ cells mL^−1^) were centrifuged and washed with PBS. The THP‐1 cells were incubated with 1 × 10^−6^ M calcein‐AM green, 2 × 10^−6^ M EthD‐1 from a live/dead viability/cytotoxicity kit (Invitrogen, L3224), and antibody‐bound beads for 40 min on a vortex rotator at room temperature for size based separation experiment. The MCF‐7 cells were also incubated with 2 × 10^−6^ M calcein‐AM green and antibody‐bound beads for 40 min on a vortex rotator at room temperature for cell clustering experiment. The fluorescent‐labeled cells, bound to magnetic beads, were then separated from unbound cells using an external magnet. Finally, the cells bound to the beads were resuspended in a fresh culture medium for the experiment.

### Statistical Analysis

For magnetic bead dynamic experiments, the data were presented as mean values ± standard deviations (SDs) for above 20 beads to obtain reliable statistics. Separation efficiency was calculated as below in Figure 5b and Table S2 (Supporting Information).

(9) $$\begin{array}{c} \mathrm{Separation}\;\mathrm{efficiency}=\frac{\mathrm{target}\;\mathrm{micro}-\mathrm{objects}\;\mathrm{in}\;\mathrm{desired}\;\mathrm{area}\;}{\mathrm{Total}\;\mathrm{target}\;\mathrm{micro}-\mathrm{objects}\;\mathrm{in}\;\mathrm{sample}\;}\times 100\;\;\% \end{array}$$

Size measurement of single cells was carried out MATLAB Image processing in Figures 6 and 7 and Figure S13 (Supporting Information). Statistical analysis and data processing were carried out MATLAB (MathWorks).

## Conflict of Interest

The authors declare no conflict of interest.

## Supporting information

Supporting Information

Supplemental Movie 1

Supplemental Movie 2

Supplemental Movie 3

Supplemental Movie 4

Supplemental Movie 5

## Data Availability

Research data are not shared.
