# Supplementary material for: Magnetophoretic Micro‐Distributor for Controlled Clustering of Cells
Source: Adv Sci (Weinh). 2021 Dec 15;9(6):2103579. doi: 10.1002/advs.202103579 (PMC8867205; doi:10.1002/advs.202103579)
Supplement: Supplementary file 1 — Supporting Information [file ADVS-9-2103579-s005.pdf]

ADVANCED SCIENCE
Open Access

Supporting Information

for *Adv. Sci.*, DOI: 10.1002/advs.202103579

Magnetophoretic micro-distributor for controlled clustering of cells

Jonghwan Yoon, Yumin Kang, Hyeonseol Kim, Sri Ramulu Torati, Keonmok Kim, Byeonghwa Lim, and CheolGi Kim**

Supporting Information

Magnetophoretic micro-distributor for controlled clustering of cells

Jonghwan Yoon, Yumin Kang, Hyeonseol Kim, Sri Ramulu Torati, Keonmok Kim, Byeonghwa Lim, and CheolGi Kim**

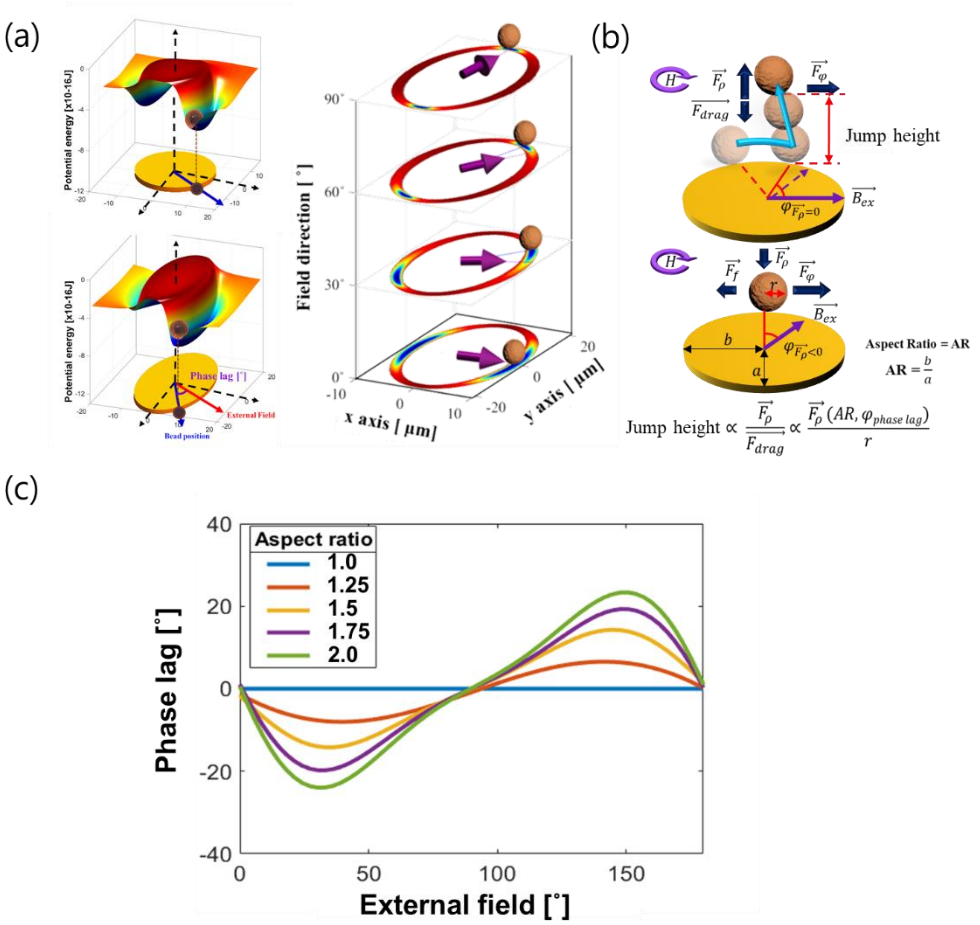

Figure S1. Phase lag of different aspect ratios of ellipse-shaped micromagnet depending on the non-uniform magnetic potential well. (a) Phase lag produced by the ellipse-shaped micromagnet under in-plane magnetic field along 30 degrees and comparing disk-shaped micromagnet. (b) Schematic of forces and variables related to bead jumping motion on the ellipse-shaped micromagnet where the aspect ratio is defined as AR (b/a) (c) Phase lag is varying with an aspect ratio of ellipse-shaped micromagnet under rotating magnetic field

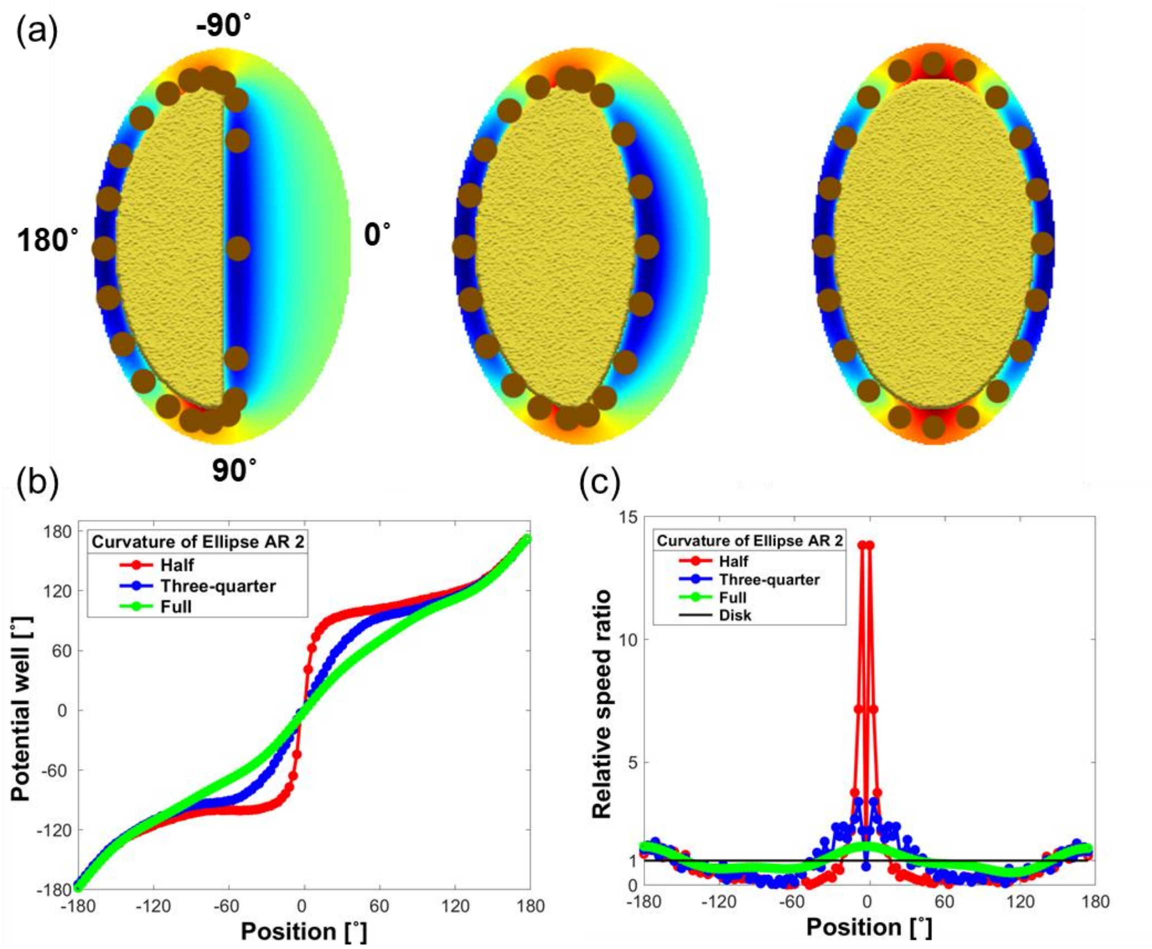

Figure S2. the stroboscope image of the magnetic potential well along Three types of Ellipse AR of 2 micromagnet under the rotation magnetic field and Potential well position and the difference in the speed of relative motion of the magnetic potential well according to different curvature of micromagnet.

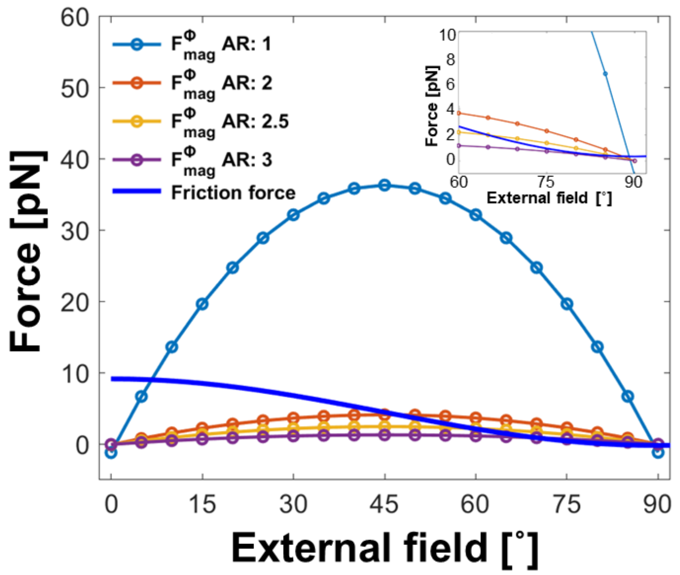

Figure S3. External field direction dependence of the F^{Φ}_{mag} which are tFhe forces received by a magnetic bead (D2.8μm) in the 0 deg position on Trampoline micromagnet with AR ranges from 1 to 3. and Friction force between the substrate and the micro-object.

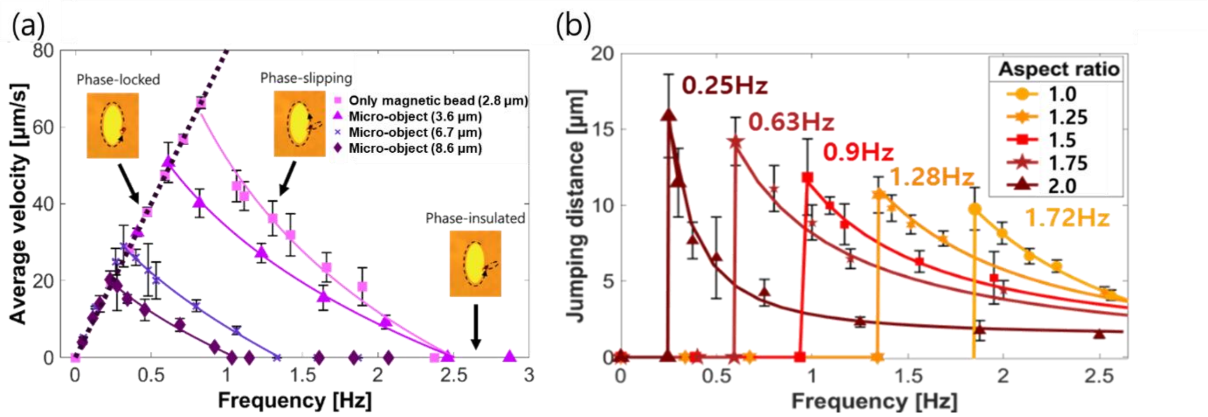

Figure S4. Dynamics of magnetic beads and magnetically labeled polymer beads on ellipse-shaped micromagnet and phase diagram of the size-based separable region using jumping motion at trampoline micromagnet according to the frequency of the external magnetic field. (a) Comparison of average velocity change based on the size of labeled polymer beads which is experimentally measured at AR 2 in 100 Oe rotating magnetic field. There are three motion phases according to the frequency of the external magnetic field (phase-locked, phase slipping, phase insulated). (All experiments were averaged for 20 beads to obtain reliable statistics) Their average velocities and critical frequencies are reduced by increased jumping distance and number of jumps which are caused by viscosity force with the increased size of the labeled polymer beads. (b) Comparison of jumping distance change at the various AR using magnetically labeled 3.6μm diameter polymer beads. This experimental data shows that the critical frequency which is labeled in the graph decreases while jumping distance increases as the AR increases. .(All experiments were averaged for 20 beads to obtain reliable statistics)

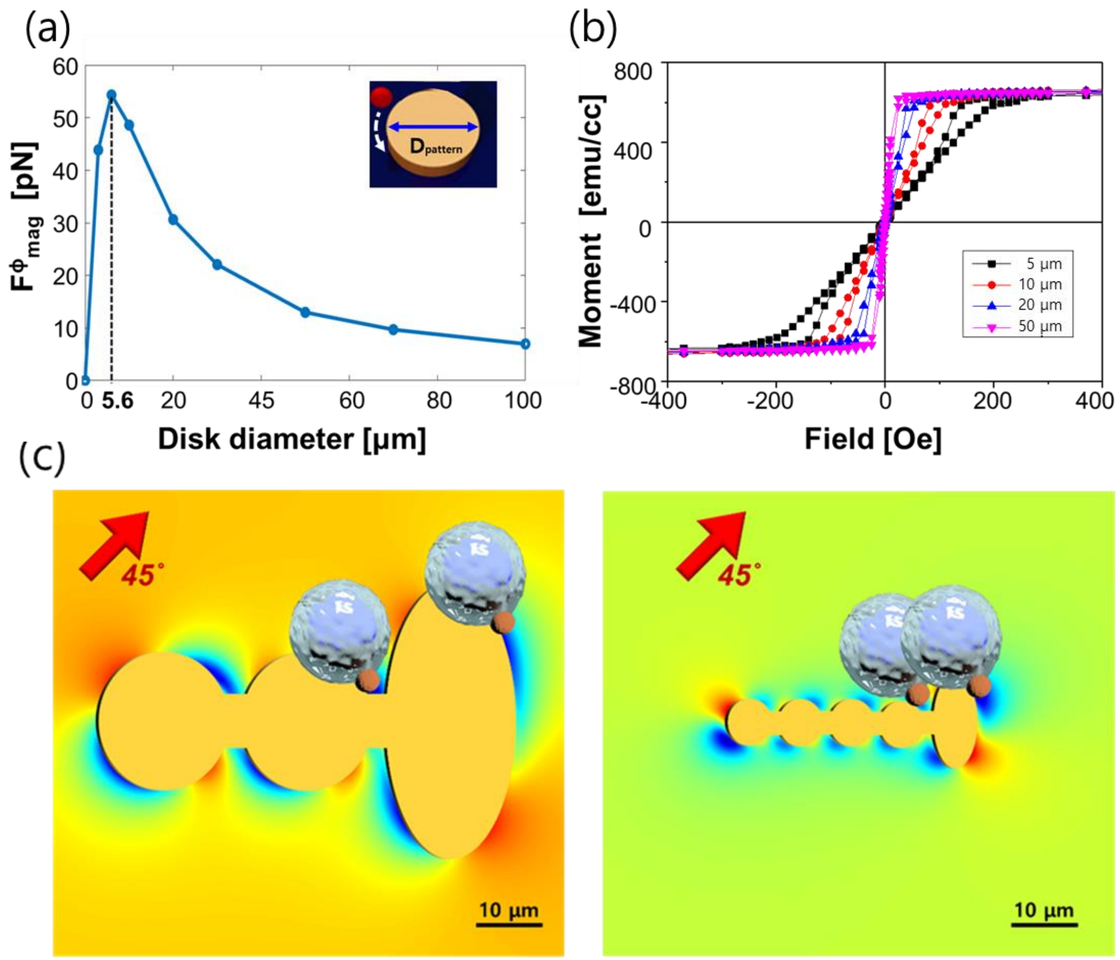

Figure S5. (a-b) φ-directional magnetic force on the bead of 2.8 μm diameter at φ=π/4 as a function of pattern radius using simulated and measured M-H initial curve of $Ni_{80}Fe_{20}$ disk micromagnet according to its size.[S1] (c) comparison of the location of the micro-objects, for example 13μm size single cells, at different magnetic potential well according to a various size (D20 μm/ D6 μm) of trampoline micromagnet under 45˚ external magnetic field.

We analyzed each element from a magnetic/physical perception to optimize the platform for the precise manipulation of 3-20 μm size micro-object.

As a magnetic perception, the magnetic force on the magnetic bead is determined by the interaction between the magnetic bead and micromagnet. Based on the numerical analysis, the obtained Figure S5-a shows that φ-directional magnetic force on the bead was maximum when the diameter of the magnetic pattern was about twice the diameter of the bead. In addition, the strength of the saturated magnetic field can be changed according to the diameter of the micromagnet as shown in Figure S5-b.[S1] and the larger the pattern, the micromagnet's maximum force can be used in a smaller magnetic field. For example, in the case of a disk magnet of 20 μm, the maximum magnetization value is reached at a field strength of about 100-150 Oe. Since we used a two-axis coil magnetic field generator that forms a uniform magnetic field up to 150 Oe ± 1 Oe in 1cm x1cm area, the limit of the pattern size for the largest magnetic force in the magnetic field range is 10-20 μm. Hence, magnetic beads with a diameter of about 5-10 μm can form the maximum magnetic force according to the Fig. S5 a-b.

As a physical perception, magnetic beads and patterns are limited in size because of issues such as fluid resistance and contact adsorption. In the case of magnetic beads, the size of the magnetic beads must be smaller than the minimum size of the target to proceed with the

dynamic analysis based on the size difference of the target cells. In this study, 3-20 µm cells were targeted, so magnetic beads with a diameter of 2.8 µm which are smaller than the 3.6 µm diameter of the smallest micro object. In addition, when cells are moved along the direction of a magnetic field in the subsequent array structure as shown in figure S5-c, each cell is manipulated by being trapped in a periodically repeated potential well. At this time, if the gap between the potential wells is smaller than the cell size, the cells are in physical contact. For individual control of single cells, it is necessary to prevent distortion due to cell contact and non-specific adhesion it should be at least 20 µm, which is the maximum size of the cells because the spacing between wells is the same as the diameter of the pattern.

Therefore, considering both magnetic and physical aspects, a pattern with a diameter of 20 µm should be used to minimize intercellular interference while creating the maximum magnetic force in the currently equipped experimental environment.

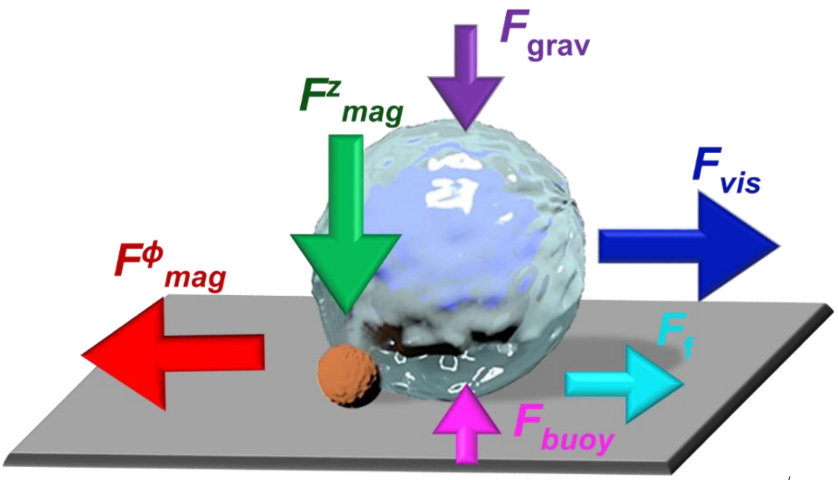

Figure S6. Schematic representation of micro-object undertaking the governing forces F^{ϕ}_{mag}, F^{z}_{mag}, F_{vis}, F_{f}, F_{grav}, and F_{buoy} denoting the tangential, vertical, viscous, frictional forces, gravitational forces and buoyancy forces, respectively, which act on a magnetic bead and labeled single cell around micromagnet in liquid environment.

	F^{ϕ}_{mag} max	Viscous force (0.65Hz)	Viscous force (0.2Hz)	F^{z}_{mag} max	Gravitational forcce	Buoyant force
**Bead (M280)**	36.84 pN	3.69 pN	1.13 pN	213.11 pN	0.16 pN	0.11 pN
**THP-1 cell**	36.84 pN	30.60 pN	9.42 pN	0 pN	18.90 pN	17.66 pN
**MCF-7 cell**	36.84 pN	35.70 pN	10.98 pN	0 pN	27.25 pN	24.33 pN

Table S1. Magnetic, viscous, gravitation, and buoyant forces which are respectively received by magnetic beads and labeled two types of the single cell.

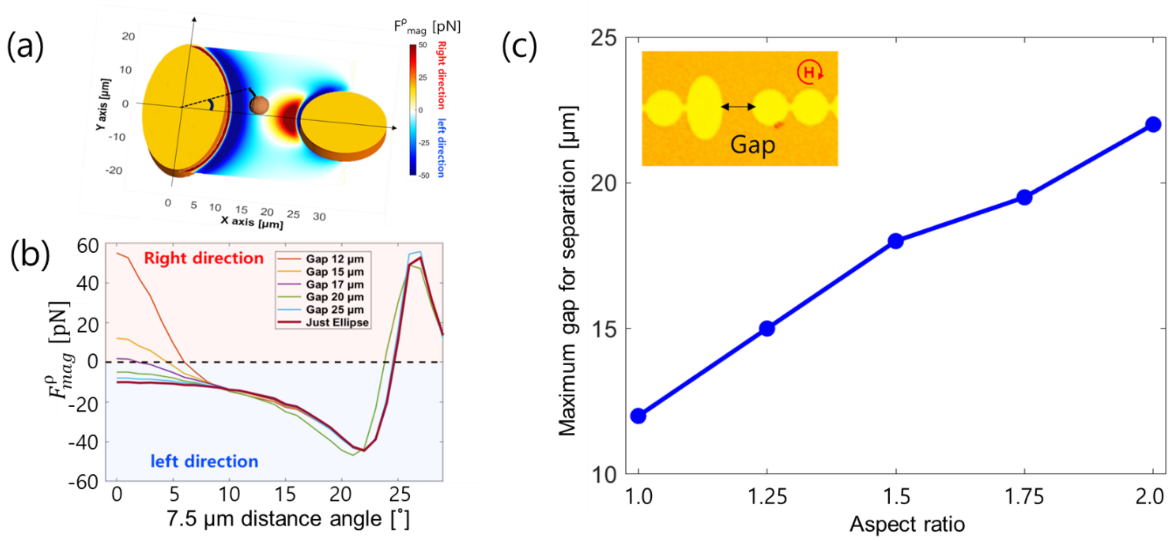

Figure S7. Separable frequency according to gap of micro-distributor. (a) Radial magnetic force landscape on AR of 2 micro-distributor of gap 15 μm. (b) The dependence of radial magnetic force on the gap of AR of 2 micro-distributor at 7.5 μm position. (c) Maximum gap for separation of micro-object (8.6 μm) according to AR on micro-distributor

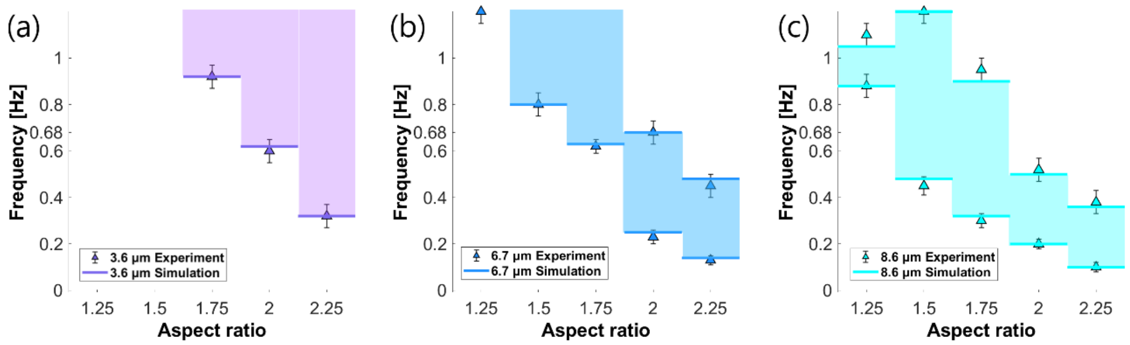

Figure S8. Comparing experiment separation conditions with the specific frequency region calculated by numerical simulation according to the size of the micro-object (3.6 μm/ 6.7 μm/ 8.6 μm) and AR of trampoline micromagnet. (All experiments were averaged for 20 beads to obtain reliable statistics)

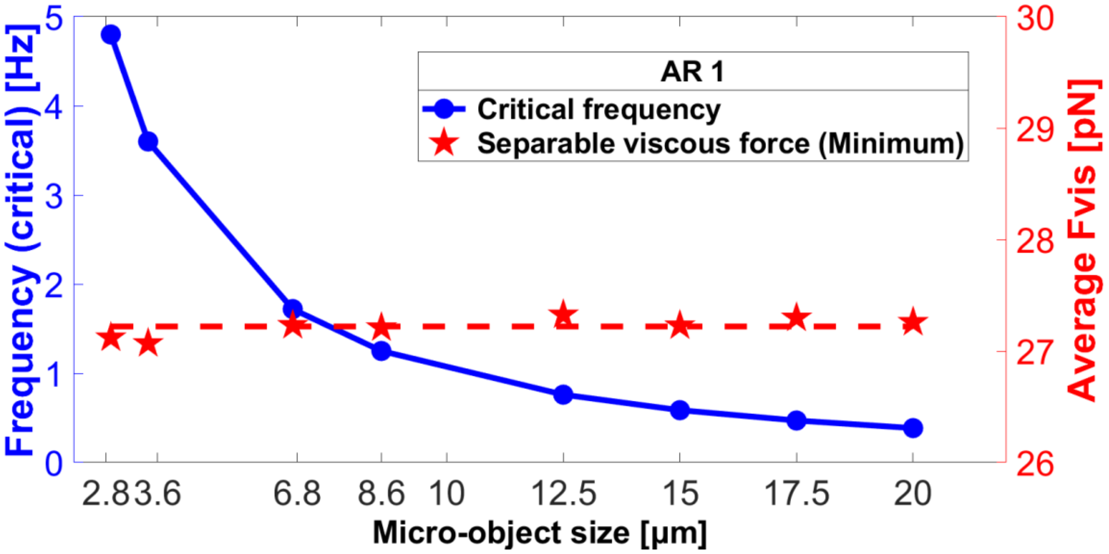

Figure S9. Comparison of the critical frequency of magnetic bead with viscous force which micro-object received per 1 cycle of the external rotating field according to micro-objects size in AR of 1 Micromagnet.

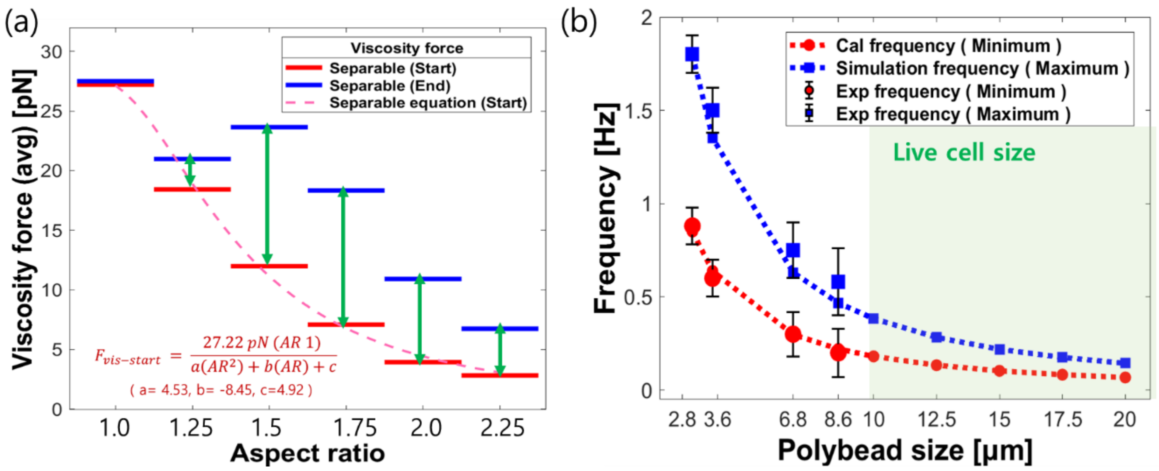

Figure S10. Calculation of separable ARs micro-distributor according to average viscous force depending on the size of the micro-object and frequency. (a) Using trajectory simulation, an average viscous force which micro-object received per 1 cycle of the external rotating field under the separable condition was obtained according to AR and obtain relational expression as the red maker Equation 6. (b) Comparison of calculated conditions based on Equation 7 with experimental conditions for separable frequency regions according to micro-objects size in AR of 2 Micro-distributor of gap 15 μm. (All experiments were averaged for 25 beads to obtain reliable statistics)

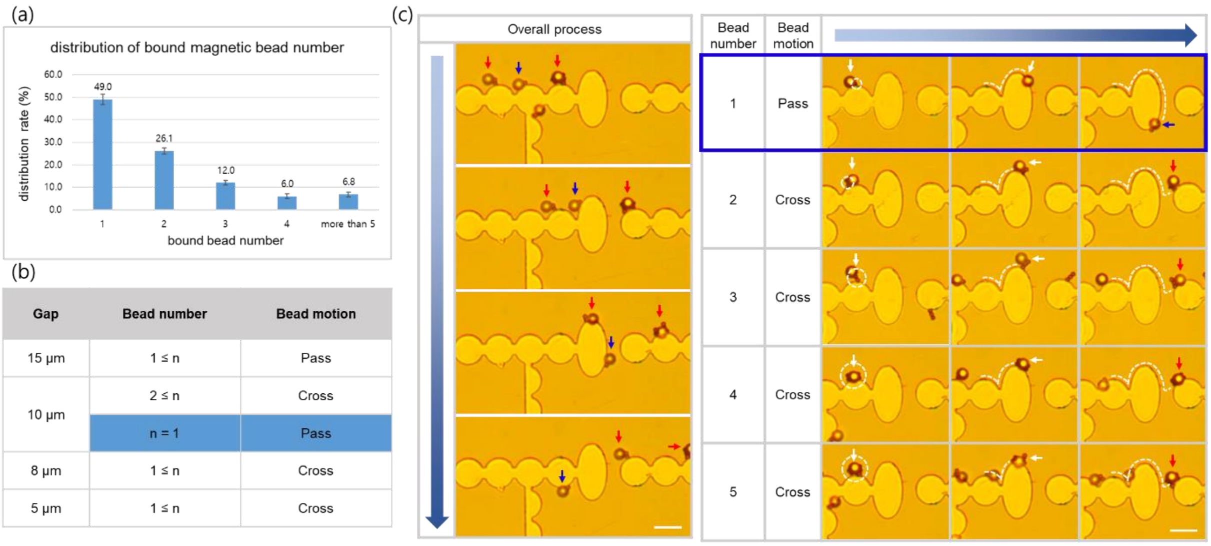

Figure S11. (a) Distribution ratio of magnetic bead bound on the micro-object. The number of magnetic beads were investigated which is bounded on the each micro-object. 8.6 μm of diameter micro-objects were used. According to the examination of 467 micro-objects, micro-objects with one magnetic bead were occupied 49% and micro-objects with more than one magnetic beads were distributed 51% and (b-c) Pre-processing circuit for separating only one magnetic bead bound micro-objects. At low frequency (0.05 Hz) of magnetic field, more than one magnetic beads bound micro-objects are cross the gap between trampoline micromagnet with AR of 2 and landing micromagnet depending on the gap size. When the gap is 10 μm, only one bead bound micro-objects pass along the trampoline micromagnet and other micro-objects cross toward the landing micromagnet because of their high magnetization induced by beads. If the gap is smaller than 10 μm, ever labeled micro-objects cross the gap.(scale bar is 20 μm)

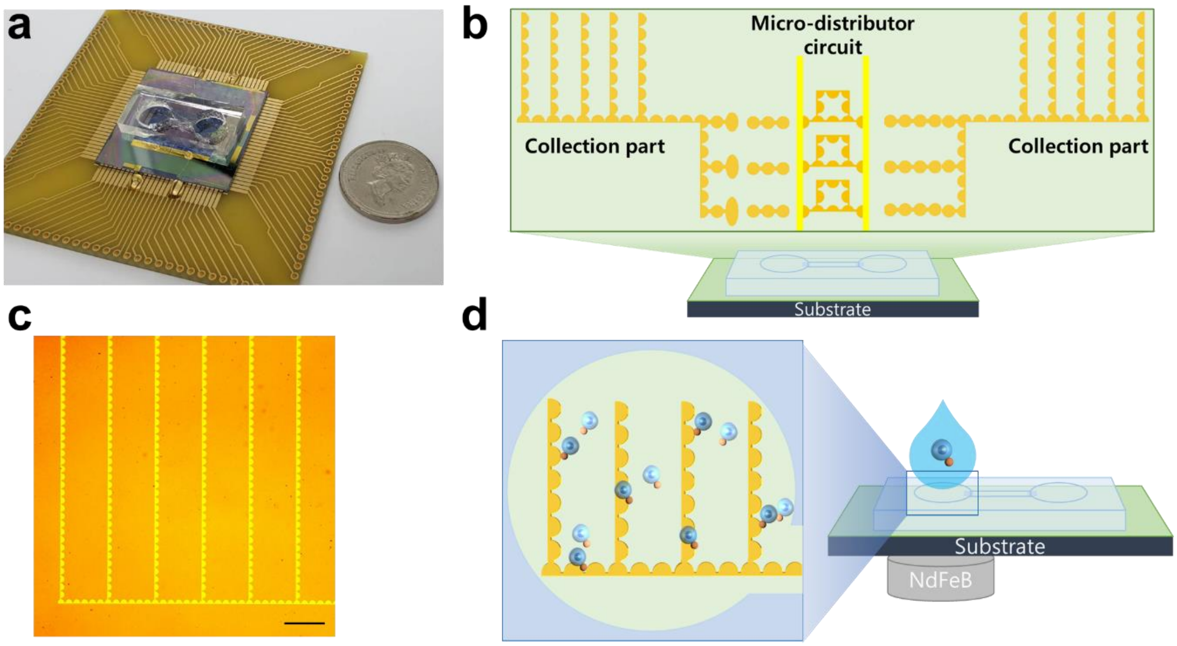

Figure S12. (a) Image of cell clustering platform device including (b) all components of micro-distributor circuit (c) collection micromagnet part. (scale bar is 50 μm) and (d) Schematic of loading process on the collection micromagnet.

Experiment & Numbers	Distribution ratio [%]			Separation efficiency [%]
	Outlet 1	Outlet 2	Outlet 3	
1 (n=49)	76.92	83.33	91.67	85.71
2 (n=100)	71.43	95.24	100	88.89
3 (n=107)	70.83	80.00	86.79	81.31
4 (n=42)	85.00	90.91	100	88.10
5 (n=37)	73.33	100	100	89.19
6 (n=26)	91.67	100	85.71	88.46
7 (n=175)	92.86	85.48	94.12	88.68
8 (n=35)	80.00	100	94.44	94.29
9 (n=42)	100	87.50	100	95.24

Table S2. Mixture numbers which are three types of micro-objects and the distributions ratios of 3 sizes of micro-objects were collected at each outlet position 1, 2, and 3 with an efficiency

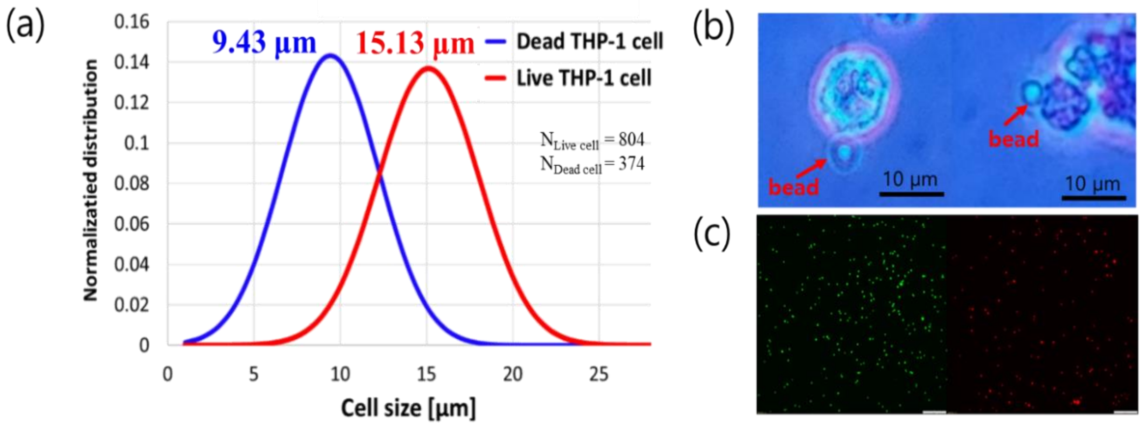

Figure S13. Size distribution of live & dead cells of THP-1 cells. (a) Size distribution of live & dead cells of THP-1 cells. Live cells had a diameter of 15.1 (± 5.2) μm in 804 samples, while dead cells that died in reaction with Camptothecin, a drug that causes Apoptosis Volume Decease (AVD), had a diameter of 9.4 (± 6.5) μm in 374 samples. (b) Live & dead cells of THP-1 cells image using Trypan blue stain. (c) Fluorescent image of live & dead cells of THP-1 cells.

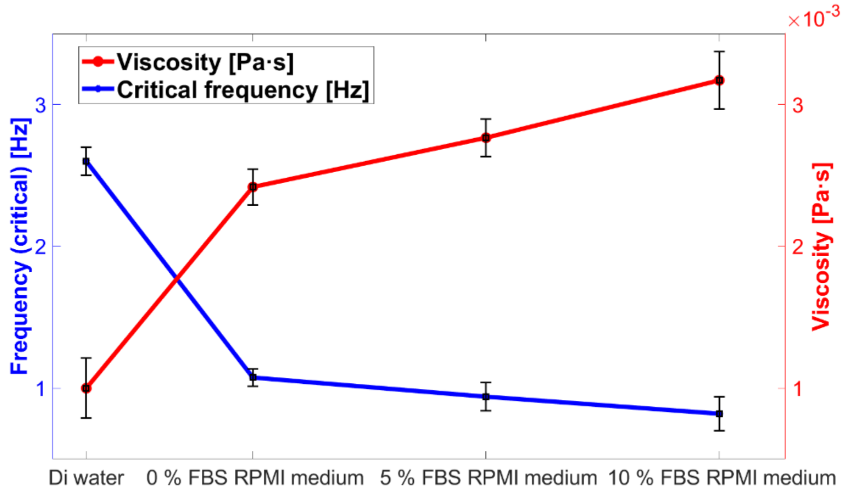

Figure S14. Critical frequency and viscosity of magnetic beads in each different cell culture environment. The critical frequency of magnetic bead on top of AR of 1 micromagnet depends on the FBS concentration and media that affect the cell culture environment. Based on the measured critical frequency, viscosity can be calculated in each environment against the viscosity of Di-water with 1 Pa·s.

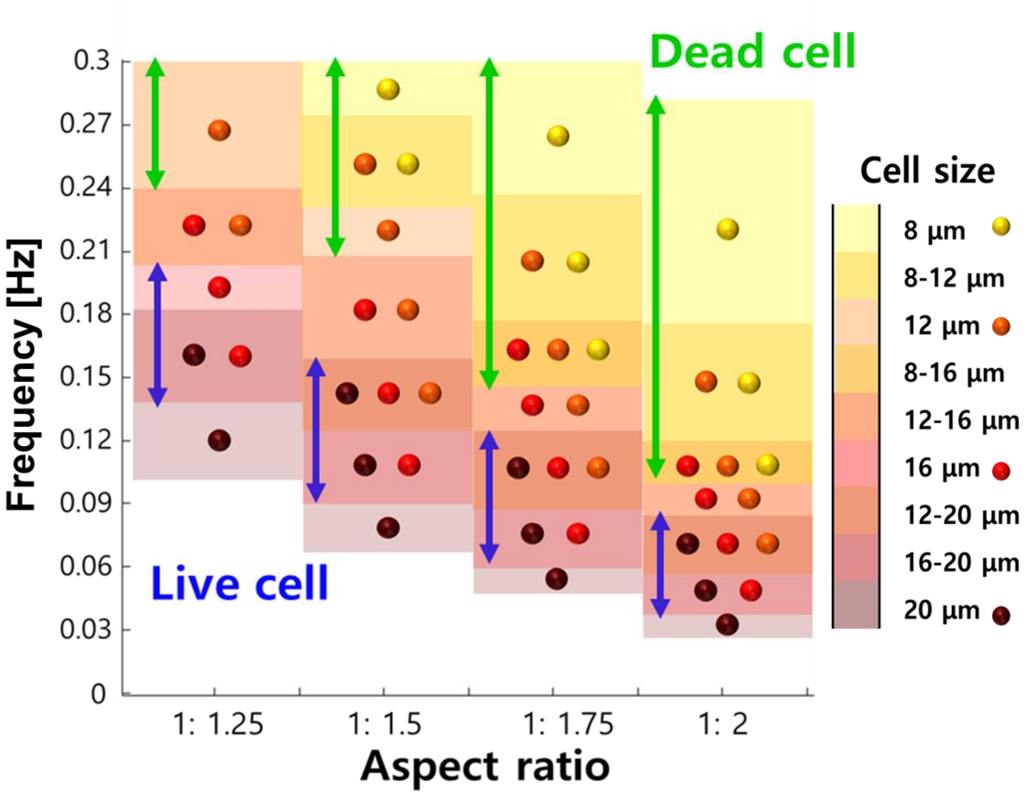

Figure S15. Phase diagram of size-based separable condition at micro-distributor according to the frequency of rotational magnetic field in a cell culture environment at 130Oe magnetic field. Separation condition of live cells sizes are blue arrow region and separation condition of dead cells sizes are green arrow region

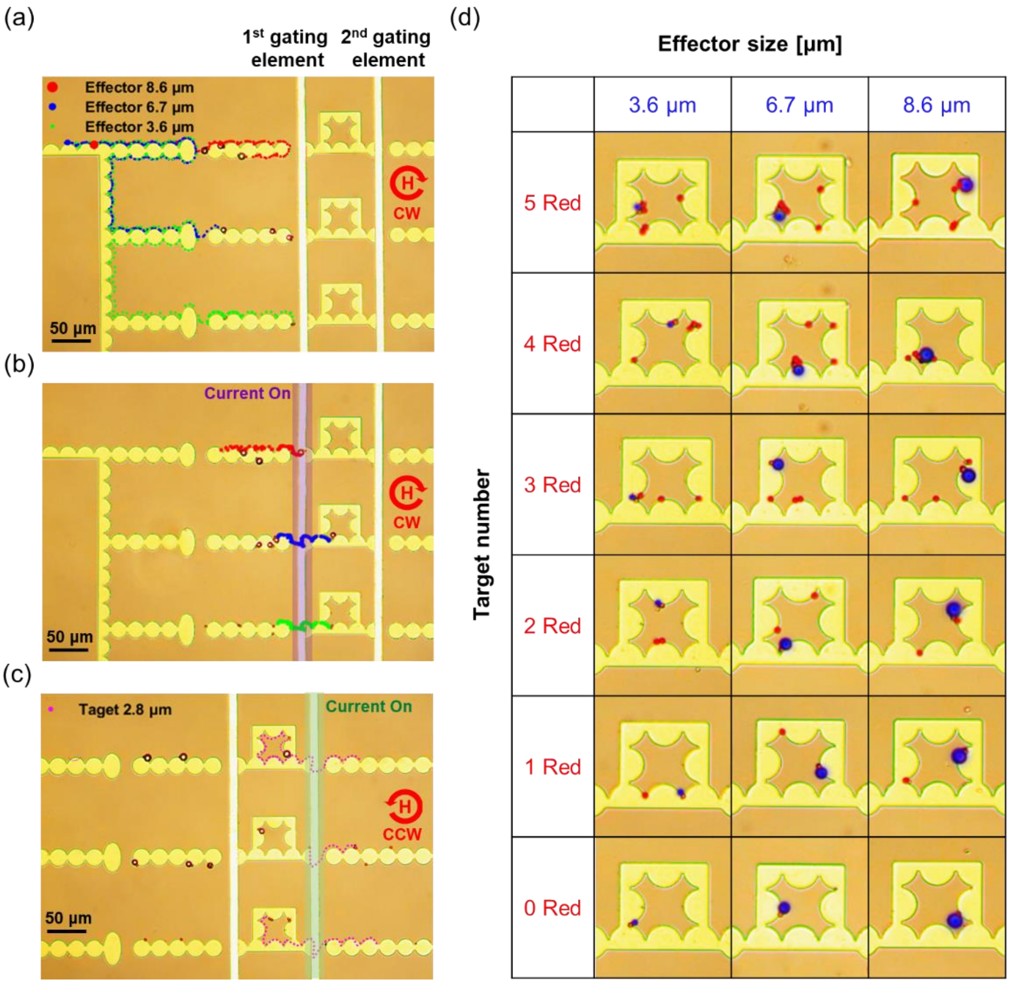

Figure S16. Micro-distributor clustering performance. Different transition trajectories pathways were obtained for micro-objects of different sizes considered as effector cells and multiple magnetic beads considered as target cells using a micro-distributor circuit for cells clustering. (a-c) The overall process for size-based separation and clustering. Size-based distribution of micro-objects was obtained at f = 0.68 Hz in a clockwise direction at 100 Oe magnetic field. On the micro-distributor including AR of 1.5, 1.75, 2 of 3 types trampoline micromagnet. (d) A table of the various combination according to the size and number of micro-objects. The micro-object and magnetic bead were considered as effector cells and target cells to demonstrate the performance of the cells clustering.

Supporting Reference

[S1] B. Lim, P. Vavassori, R. Sooryakumar, C. Kim, *J. Phys. D: Appl. Phys*. **2017**, 50 033002

Supporting Movies

Movie S1: Three different sizes of micro-objects sorting procedure on the micro-distributor

Movie S2: Two different sizes of THP-1 live cell and dead cell sorting procedure & Two different sizes of THP-1 live cells sorting procedure on the micro-distributor

Movie S3: Three different sizes of effector automatically separation procedure on micro-distributor

Movie S4: The quantitative separation procedure for the target beads into each clustering room using 2nd gating element

Movie S5: Cell-clusteting procedure for the THP-1 cell and MCF-7 cells into each clustering room
